# Advanced Fluorescence Microscopy Techniques—FRAP, FLIP, FLAP, FRET and FLIM

**DOI:** 10.3390/molecules17044047

**Published:** 2012-04-02

**Authors:** Hellen C. Ishikawa-Ankerhold, Richard Ankerhold, Gregor P. C. Drummen

**Affiliations:** 1 Ludwig Maximilian University of Munich, Institute of Anatomy and Cell Biology, Schillerstr. 42, 80336 München, Germany; 2 Carl Zeiss Microimaging GmbH, Kistlerhofstr. 75, 81379 München, Germany; 3 Bionanoscience and Bio-Imaging Program, Cellular Stress and Ageing Program, Bio&Nano-Solutions, Helmutstr. 3A, 40472 Düsseldorf, Germany

**Keywords:** fluorescence microscopy, fluorescence, fluorochrome, techniques, confocal, multiphoton, anisotropy, FRET, homo-FRET, FRAP, FLIP, FLIM, FLAP

## Abstract

Fluorescence microscopy provides an efficient and unique approach to study fixed and living cells because of its versatility, specificity, and high sensitivity. Fluorescence microscopes can both detect the fluorescence emitted from labeled molecules in biological samples as images or photometric data from which intensities and emission spectra can be deduced. By exploiting the characteristics of fluorescence, various techniques have been developed that enable the visualization and analysis of complex dynamic events in cells, organelles, and sub-organelle components within the biological specimen. The techniques described here are fluorescence recovery after photobleaching (FRAP), the related fluorescence loss in photobleaching (FLIP), fluorescence localization after photobleaching (FLAP), Förster or fluorescence resonance energy transfer (FRET) and the different ways how to measure FRET, such as acceptor bleaching, sensitized emission, polarization anisotropy, and fluorescence lifetime imaging microscopy (FLIM). First, a brief introduction into the mechanisms underlying fluorescence as a physical phenomenon and fluorescence, confocal, and multiphoton microscopy is given. Subsequently, these advanced microscopy techniques are introduced in more detail, with a description of how these techniques are performed, what needs to be considered, and what practical advantages they can bring to cell biological research.

## 1. Introduction

FRAP, FLIP, FLAP, FRET, and FLIM are fluorescence microscopy techniques that in some way take advantage of particular aspects of the fluorescence process by which fluorochromes are excited and emit fluorescent light, are damaged during repetitive excitation, or undergo non-radiative decay prior to light emission. In order to understand the basic principles underpinning these advanced fluorescence techniques, first some general aspects of fluorescence and fluorescence microscopy are introduced before going into the technical details and practicalities of FRAP, FLIP, FLAP, FRET and FLIM. This article is not meant to be a comprehensive report on the aforementioned techniques, but rather to introduce these advanced fluorescence imaging techniques to a broad biological and bio(medical) research audience and give the reader some feeling for the field. The reader is referred to more specialized and comprehensive books and manuscripts for further reading throughout the text.

### 1.1. Introduction to Fluorescence

#### 1.1.1. The Physical Phenomenon of Fluorescence

**Figure 1:**
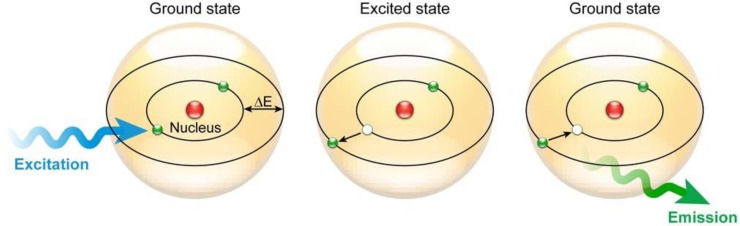
Fluorescence principle. Schematic representation of the fluorescence phenomenon in the classical Bohr model. Absorption of a light quantum (blue) causes an electron to move to a higher energy orbit. After residing in this “excited state” for a particular time, the fluorescence lifetime, the electron falls back to its original orbit and the fluorochrome dissipates the excess energy by emitting a photon (green).

Fluorescence as a phenomenon is part of a larger family of related luminescent processes in which a susceptible substance absorbs light, only to reemit light (photons) from electronically excited states after a given time (Figure 1). Photoluminescent processes that are generated through excitation, whether this is via physical, mechanical, or chemical mechanisms, can generally be subdivided into fluorescence and phosphorescence.

Compounds that display fluorescent properties are generally termed fluorescent probes or dyes and *de facto* the term ‘fluorochrome’ is most appropriate. Often ‘fluorochrome’ and ‘fluorophore’ are used interchangeably. Strictly taken the term ‘fluorophore’ refers to fluorochromes that are conjugated covalently or through adsorption to biological macromolecules, such as nucleic acids, lipids, or proteins. Fluorochromes come in different flavors and include organic molecules (dyes), inorganic ions (*e.g.*, lanthanide ions such as Eu, Tb, Yb, *etc.*), fluorescent proteins (*e.g.*, green fluorescent protein), and atoms (such as gaseous mercury in glass light tubes). Some commonly used fluorochromes are listed in Table 1. Recently, inorganic luminescent semiconducting nanoparticles, quantum dots, have been introduced as labels for biological assays, bio-imaging applications, and theragnostic purposes—the combination of diagnostic and therapeutic modalities in one and the same particle (see reference [1]).

**Figure 2:**
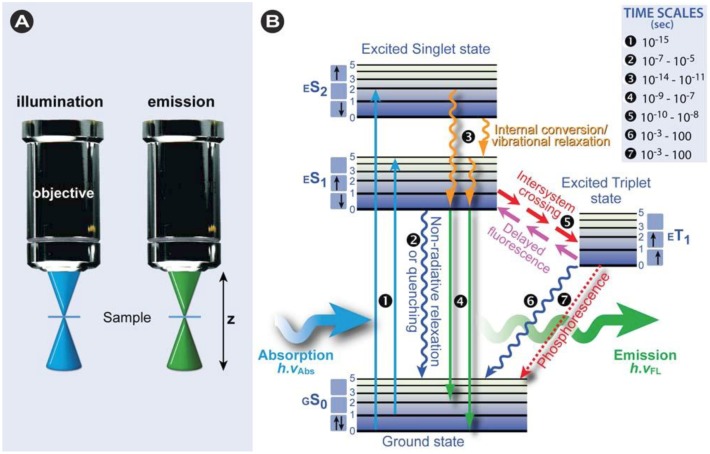
Single photon excitation. (**A**) In fluorescence microscopy the sample is illuminated with excitation light and the emitted light–the fluorescence–is detected through the same objective (*epi*-fluorescence microscopy). (**B**) Jabłoński diagram: An electron that leaves the ground state *GS*_0_ (electronic singlet) when a quantum of light (a single photon) is absorbed and moves to a higher excited state, relaxes quickly to a lower vibrational excited state (orange line) and thereby looses energy. When returning to the ground state it dissipates the remaining energy by emitting a photon with a longer wavelength, *i.e.*, fluorescence emission. The spins of electrons in the singlet states (paired or unpaired anti-parallel spins) compared to the triplet state (unpaired, parallel spin) are depicted. Notice that intersystem crossing from *ES*_1_→*ET*_1_ requires spin conversion.

Fluorescence follows a series of discrete steps of which the outcome is the emittance of a photon with a longer wavelength—a process which can be visualized in more detail via the Jabłoński diagram in Figure 2B. When light of a particular wavelength hits a fluorescent sample, the atoms, ions or molecules therein absorb a specific quantum of light, which pushes a valence electron from the ground state *GS*_0_—this initial state is an electronic singlet in which all electrons have opposite spin and the net spin is 0—into a higher energy level (Figure 1 and Figure 2B), creating an excited state *ES*_n_. This process is fast and in the femtosecond range and requires at least the energy Δ*E* = *E*_ESn_ − *E*_GS__0_ to bridge the gap between excited and ground states in order for excitation to occur. The energy of photons involved in fluorescence and generally a quantum of light can be expressed via Planck’s law [2]:


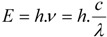
 (1)

where *E* is the quantum’s energy (J), *h* is Planck's constant (J.s), *ν* the frequency (s^−1^), *λ* is the wavelength of the photon (m), and *c* is the speed of light (m.s^−1^). However, there are several excited state sublevels (vibrational levels) and which level is reached primarily depends on the fluorescent species’ properties.

**Figure 3:**
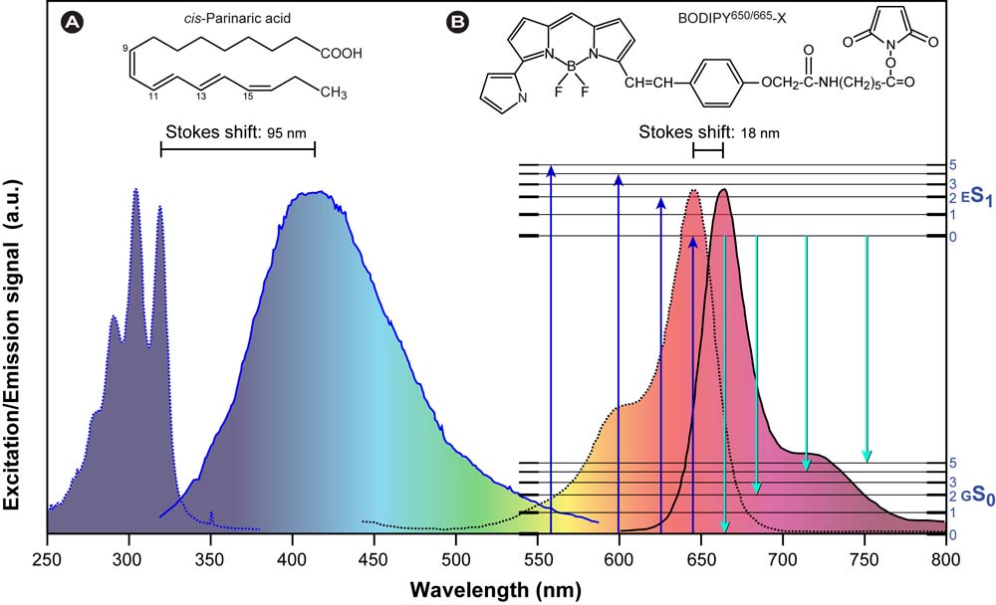
Stokes shift and mirror image rule. This diagram shows typical absorption/excitation and emission spectra of two different fluorescent dyes: (**A**) *cis*-Parinaric acid; (**B**) BODIPY^650/665^. The difference between the excitation and emission maxima is called “Stokes shift”. It is caused by a quick electron relaxation and intramolecular vibrational energy loss taking place between the different excited state sublevels. That is the reason why the excitation light normally has a shorter wavelength (*i.e.*, higher energy) than the emission light (*i.e.*, lower energy). In many fluorochromes, the same electronic transitions are involved in both excitation and emission, which leads to near-mirror image spectra. This is not always the case, as exemplified by *cis*-parinaric acid (**A**), which characteristically shows four maxima, corresponding to the four conjugated double bonds in the absorption spectrum and a broad single maximum emission spectrum.

Irradiation with a spectrum of wavelengths generates numerous allowed transitions that populate the various vibrational energy levels of the excited states, some of which have, according to the Franck-Condon principle, a higher probability to occur than others (the better two vibrational wave functions overlap, the higher the probability of transition) and combined form the absorption spectrum of the fluorescent dye (Figure 3B).

After excitation to the higher energy level *ES*_n_, the electron quickly relaxes to the lowest possible excited sublevel, which is in the picosecond range. The energy decay from dropping to a lower vibrational sublevel occurs through intramolecular non-radiative conversions and the converted heat is absorbed via collision of the excited state fluorescent molecule with the solvent molecules. Emission spectra are usually independent of the excitation wavelength because of this rapid relaxation to the lowest vibrational level of the excited state, which is known, as Kasha’s rule [3]. In most cases, absorption and emission transitions involve the same energetic levels as schematically shown in Figure 3B. For this reason and the fact that excitation is instantaneous, involving electrons only and leaving the heavier nuclei in place, many fluorochromes display near mirror image absorption and emission spectra (mirror image rule), although many exceptions exist, as exemplified by the spectrum of *cis*-parinaric acid in Figure 3. External conversion depletes the excited state through interaction and energy transfer to the solvent and/or solute. Intersystem crossing is the slowest energy dissipation pathway, because the electron has to change spin multiplicity from an excited singlet state to an excited triplet state (Figure 2B). This is essentially a spin forbidden transition, but in some fluorochromes favorable vibrational overlap between the two states makes the transition weakly allowed. Intersystem crossing is mostly observed in molecules containing heavy atoms such as iodine or bromine or in paramagnetic species.

Lanthanide ions are good examples of luminescent probes that show delayed fluorescence—The term “luminescence” is normally used when discussing lanthanide-based emission, because “fluorescence” refers to spin allowed singlet-to-singlet emission, whereas in lanthanide ions, emission is due to intra-configurational f-f transitions (transitions inside the 4f shell) [4]. As a result they have fluorescence lifetimes (the average time in the excited state) in the (sub)microsecond range for Yb(III) and Nd(III), whereas Eu(III), Tb(III) and Sm(III) have even longer lifetimes in the (sub)millisecond range [5]. This is significantly longer than the lifetimes of organic fluorochromes and fluorescent proteins which are generally in the nanosecond range [6]. Because of the large lifetime difference to fluorescence, lanthanide emission should easily be distinguishable from fluorescence emission by time-gating techniques. A critical review on lanthanides as probes can be found in [7].

When the electron finally returns to the lower energy level it originated from, the ground state *GS*_0_, a quantum of light (photon) is emitted with a longer wavelength, which is an allowed transition since the spin is retained. In most fluorochromes, photon emission is produced by a π*→π or π*→*n* transition, depending on which requires the least energy for the transition to occur. In contrast, σ*→ σ transitions are rare since the required UV light (below 250 nm) is energetic enough to deactivate the excited state electron by predissociation (internal conversion to a *GS*_0_ that is at such a high vibrational level that the bond breaks) or dissociation (the bond breaks at a vibrational excited state). 

Photon emittance from singlet states with an average time-scale between 10^−9^ and 10^−6^ seconds is slow compared with the absorption of photons. This resulting emitted radiation is called fluorescence [8] and in the simplest form of fluorescence microscopy, this emitted light is collected and transported to the detector through the same objective lens used to focus the excitation light onto the sample, as schematically depicted in Figure 2A. Fluorochromes can enter repetitive cycles of excitation and emission as long as no destruction or covalent modification occurs that irreversibly interrupts this process. The transition from the meta-stable triplet state *ET*_1_ to the ground state *GS*_0_ is, because of the necessary spin reversal, forbidden and therefore several orders of magnitude slower than fluorescence, *i.e.*, thus called phosphorescence. Consequently, the emission takes place over long periods of time—in some materials it takes several seconds to even minutes.

Because of the internal energy decay at the excited state levels and since the wavelength varies inversely with the radiative energy (Equation 1), fluorescence emission generally occurs at longer wavelengths and concomitant lower energy than the light used to excite the fluorochrome, provided that a single photon is involved during excitation. It was the British scientist Sir George Stokes who first observed fluorescence when irradiating fluorspar (fluorite) with UV radiation and a red-shift in the resulting emitted light, which he reported in his 1852 publication “on the change of refrangibility of light” [9]. The difference between the emission and excitation maxima is called “Stokes shift” as represented in Figure 3. The Stokes shift varies markedly among different fluorochromes. Additionally, it should be noted that anti-Stokes shifts in which the emission wavelength is smaller than the excitation wavelength are also possible, for instance during photon upconversion or two-photon excitation (discussed below). Anti-Stokes fluorescence also occurs commonly in fluorochromes in which absorption and emission spectra overlap substantially. In summary, fluorochromes not only have characteristic excitation spectra, but also characteristic emission spectra that are dependent on their specific vibronic configuration and properties [10,11].

#### 1.1.2. Overview of Fluorescence Characteristics

This paragraph concisely introduces some of the essential characteristics and parameters of fluorescence that are utilized in fluorescence microscopy, such as the fluorescence life time, quantum yield, quenching, photobleaching, energy transfer, and others. Some of these fluorescent properties may be experienced by one researcher as an unwanted “artifact”, *e.g.*, photobleaching or intensity loss via resonance energy transfer, whereas the same feature may be cleverly used by another to solve her/his scientific question, *e.g.*, to study diffusion of molecules via FRAP or molecular interactions via FRET.

**Figure 4:**
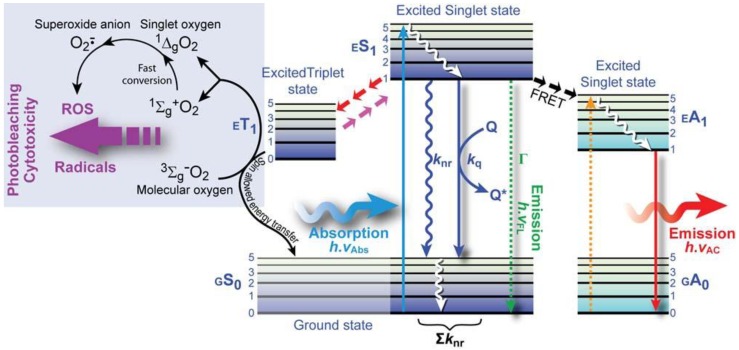
.Fluorescence deactivation mechanisms. This Jabłoński diagram shows several processes that deplete the excited state non-radiatively. From the lowest vibrational level of the excited state level *ES*_1_, several options are open for decay: (i) emission of a longer wavelength photon (*h.ν*_FL_); (ii) various processes that cause non-radiative relaxation, collectivelydenoted with rate *k*_nr_; (iii) quenching by surrounding molecules (*k*_q_), most notably dissolved molecular oxygen; (iv) Förster resonance energy transfer (FRET) to a suitable acceptor molecule that results in acceptor emission; or (v) intersystem crossing to the triplet excited state that results in the formation of reactive oxygen species (ROS) and radicals from ground state molecular oxygen or direct chemical reactions with surrounding molecules.

*Quantum yield*: The quantum yield (Ф) basically determines how bright a fluorochrome’s emission is. It is given by the ratio of the number of emitted to absorbed photons, which is determined by the rate constants of emission (Γ) and the sum of all non-radiative decay processes (*k_nr_*) that depopulate the excited state (Figure 4). These decay processes, such as intersystem crossing (*k_isc_*), internal conversion (*k_ic_*), predissociation (*k_pd_*), dissociation (*k_d_*), and external conversion (*k_ec_*), affect the fluorescence outcome, including quantum yield. The fraction of fluorochromes that dissipate the absorbed energy through emission can thus be written as (0 < Ф ≤ 1):


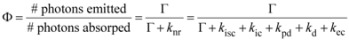
 (2)

The quantum yield, as many intrinsic fluorescence parameters, is sensitive to environmental influences, such a solvent polarization, pH, fluorochrome concentration, and the presence of molecules affecting the excited state, such as molecular oxygen. The most reliable method for recording Ф is via the comparative method established by Williams *et al.* [12], which involves comparing the fluorochrome with well characterized standard samples with known Ф values. Please note that the quantum yield is sometimes incorrectly termed ‘quantum efficiency’, which refers to the efficiency by which photons hitting a photo-reactive surface will produce electron–hole pairs in photo-sensitive devices, such as a charge-coupled device (CCD) or solar cells. 

*Fluorescence lifetime*: The fluorochrome’s fluorescence lifetime (τ) is the average time the electron spends in the excited state before returning to the ground state. In other words, the depopulation of excited state molecules through radiative (Γ) and non-radiative processes (*k_nr_*) follows an exponential decay and the time of this process is basically the fluorescence lifetime:


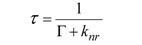
 (3)

The fluorescence intensity decay (*I_t_*) over time (t) following an infinitesimally short excitation (δ-type) may thus be expressed as:


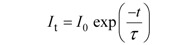
 (4)

where *I_0_* is the initial intensity. During the lifetime, the fluorochrome may undergo conformational changes, diffuse, or interact with surrounding molecules, offering an opportunity to exploit lifetime measurements to probe such actions.

*Anisotropy*: This refers to the quality of having different properties along different axes: in a pool of randomly oriented fluorochromes, only those fluorochromes with transition dipole moments that are aligned (near) parallel to the polarization direction of the excitation beam (linearly polarized) will be excited (photoselection). Fluorescence polarization is determined by the orientation of the fluorochromes’ transition moment at the instant of emission, which allows the determination of the fluorochromes’ rotation by measuring their anisotropy. Theoretically, the maximal anisotropy is achieved when the emission transition dipole moment is exactly parallel to the absorption transition dipole moment.

*Fluorescence quenching:* Quenching is a phenomenon by which interaction of a molecule, the quencher, with the fluorochrome reduces the quantum yield or the lifetime (Figure 4). Quenching phenomena can be subdivided into:

➲ Dynamic quenching occurs through collision of the quencher and the excited state fluorochrome, which leads to a decrease in the lifetime and emission intensity.
➲ Static quenching arises from direct interaction of the fluorochrome and quenching molecules, for instance by forming a non-fluorescent ground state complex. This form of quenching does not necessarily decrease the measured emission lifetime and often occurs simultaneous with dynamic quenching.
➲ In self-quenching (concentration quenching), the fluorochrome quenches its own fluorescence because of close proximity of identical molecules at high concentration. Various mechanisms underlie self-quenching, including radiationless energy transfer–this occurs particularly in fluorochromes with small Stokes shifts–or formation of molecular aggregates. Self-quenching occurs in particular in biomembranes, where the lipid bilayer behaves as a two dimensional fluid with different domains of fluidity where fluorochromes can be concentrated or when labeling proteins with multiple labels.
➲ Color-quenching is a process in which emitted photons are absorbed by a strongly colored component such as β-carotene. This leads to a decrease in intensity, but not the fluorescence lifetime.

*Fluorescence intermittency or “blinking”*: This phenomenon occurs when the fluorochrome randomly alternates between a fluorescent (“on”) and dark state (“off”) despite continuous excitation illumination. As a result, when tracking single fluorochromes, a stroboscopic effect may interfere with the tracking procedure. Blinking generally follows power law statistics and occurs commonly in luminescent nanoparticles, but also in some organic dyes, and fluorescent proteins. The random nature and power law dynamics generally frustrates and precludes comparison of results between independent experiments, because the “on” and “off” time distributions can significantly differ.

*Autofluorescence:* Fluorescence that does not originate from the fluorochrome of interest (FOI), but rather from cellular components that have fluorescent properties (background fluorescence), most notably flavins, *e.g.*, FAD^+^ and riboflavin, NAD(P)H, and extracellular matrix components, *e.g.*, elastin and collagen, but also retinol, folic acid, lipofuscin, and chlorophyll. The autofluorescence window approximately encompasses the range from 350 to 600 nm and can effectively be avoided by a number of strategies [13,14]: (i) precise filtering of the FOI signal with narrow bandpass optical filters, (ii) the use of probes that fluoresce outside the autofluorescence window, commonly in the (near) infrared ([N]IR) region, (iii) the use of time-resolved techniques (autofluorescent biofluorochromes have distinctly different lifetimes), (iv) the use of upconverting fluorochromes that allow excitation in the IR; (v) multispectral imaging (recording spectra in every pixel), and (vi) spectral unmixing (mathematical disentangling of mixed emission signals).

*Photobleaching*: Photobleaching or “fading” is a photochemical process in which the fluorochrome’s ability to enter repetitive excitation/emission cycles is permanently interrupted by destruction or irreversible covalent modification of the fluorochrome by reaction with surrounding (bio)molecules. Photodestructive and photochemical processes occur predominantly when the fluorochrome is in the dark triplet excited state, which is longer lived than singlet states and exhibits a high degree of chemical reactivity (Figure 4). Predominantly, reactions of the triplet state with molecular oxygen, which is a triplet state biradical (parallel spin) in the ground state, causes transformation to a reactive singlet state, *i.e.*, singlet oxygen, formation of the superoxide anion radical and other reactive oxygen species (ROS), These oxidizing ROS not only bleach the fluorochrome, but are also cytotoxic to cells, because they irreversibly modify biomolecules.

*Resonance energy transfer:* This is a photophysical process in which the excited state energy from a donor fluorochrome is transferred via a non-radiative mechanism to a ground state acceptor chromophore via weak long-range dipole–dipole coupling (Figure 4). The theoretical basis for the molecular interactions involved in resonance energy transfer was first described by Theodor Förster in the 1940s [15,16], and requires that the donor’s emission spectrum overlaps the acceptor’s absorption spectrum and that donor and acceptor are in close proximity. This provides the foundation for FRET microscopy, as discussed in more detail below.

*Charge-transfer complexes*: These are nanosecond short-lived homodimers (excimer) or heterodimers (exciplex) of two molecules of which at least one is in the excited state that show red-shifted emission compared with the monomer’s emission. Such complexes occur via electrostatic attraction because of partial electronic charge transfer between the individual entities. The individual ground state monomers would normally not associate and the monomers in the complex dissociate and repel each other once relaxation to the ground state occurs. Since these complexes require close proximity, excimer and exciplex formation require high concentrations of the monomers.

Many different fluorescent techniques have since been developed that utilize the specific mechanisms involved in fluorescence, from the initial excitation to the emission of the photon, whether this is by exploiting the differences in fluorescence lifetime, *e.g.*, distinguishing the fluorochrome’s signal from auto-fluorescence background or using the lifetime to determine molecular interactions as used in FLIM-FRET, or to use the fading of the signal as a method for measuring molecular diffusion as in FRAP. This review focuses on photobleaching and energy transfer-based microscopic techniques, which are discussed further below.

### 1.2. Fluorescence Microscopy

#### 1.2.1. General Concepts

The first fluorescence microscopes were developed at the beginning of the 20th century. In 1904, August Köhler constructed an ultraviolet (UV) illumination microscope at Zeiss Optical Works in Jena, Germany, in which he used a cadmium arc lamp as a light source. However, it was Oskar Heimstädt who developed the first working fluorescence microscope in 1911, with which he studied autofluorescence in organic and inorganic compounds [17]. Still, these early fluorescence microscopes suffered from several drawbacks, including the fact that the light sources at that time did not have enough power to excite fluorochromes at high enough rates and that effective separation of the fluorescence signal from the excitation light was difficult to achieve. In order to obtain sufficient signal, Heimstädt had to rely on darkfield illumination, which ensured that only a limited amount of excitation light would enter the objective lens, but this was a technically challenging method and certainly not suited for developing fluorescence microscopy as a mainstream tool. The invention of the *epi*-fluorescence microscope in 1929 by Philipp Ellinger and August Hirt was a major step forward to achieving this goal. 

**Figure 5:**
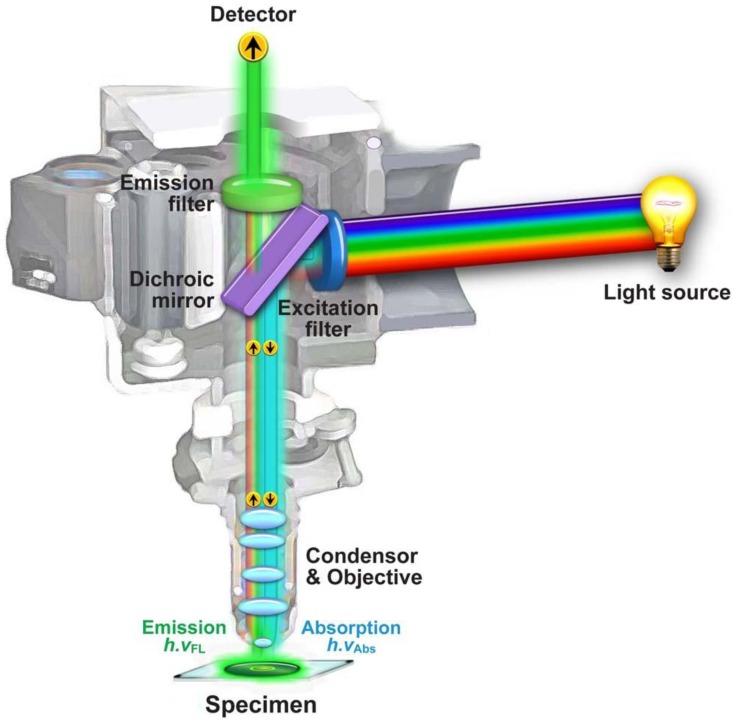
Anatomy of an *epi*-illumination fluorescence microscope.

In this configuration the illumination and detection takes place from one side of the sample (Figure 5), thereby ensuring that only reflected excitatory and emitted light reach the objective, which results in a significantly improved signal to noise ratio. In the 1930s the Austrian Max Haitingen and others developed the technique of ‘secondary fluorescence’ in which samples were systematically stained with fluorescent dyes, initially simply to make the weak autofluorescent biological samples better visible. Haitingen also was the first to introduce the word ‘fluorochrome’ for these fluorescent stains. In the early 1940s, Albert Coons developed the technique of labeling antibodies with fluorescent dyes [18]. This was a breakthrough, as it allowed to specifically label proteins and subcellular structures and as a result made these molecular structures visible in a contrast and resolution never seen before. Since these first experiments, antibody staining with fluorescent secondary markers has become a standard method in biological and biomedical research, and clinical diagnostics for fixed samples, including tissues and single cells. Furthermore, a significant increase in fluorescence signal can be achieved with these fluorochromes over the weak autofluorescent endogenous biological species. The lack of excitation power was resolved with the development of lasers in the 1960s based on Einstein’s theoretical foundations regarding stimulated emission [19] by Gould, Townes, Schawlow, and Maiman [20,21]. Lasers offered what other light sources could not: a high degree of spatial and temporal coherence, which means that the diffraction limited monochromatic and coherent beam can be focused in a tiny spot, achieving a very high local irradiance. Furthermore, it now became possible to effectively separate signals by using suitable filters and dichroic mirrors (also called dichromatic beamsplitters). The latter are specialized interference filters that selectively allow passage of light in a particular wavelength range, while reflecting other wavelengths when placed into the light path at a 45° angle (Figure 5).

**Figure 6:**
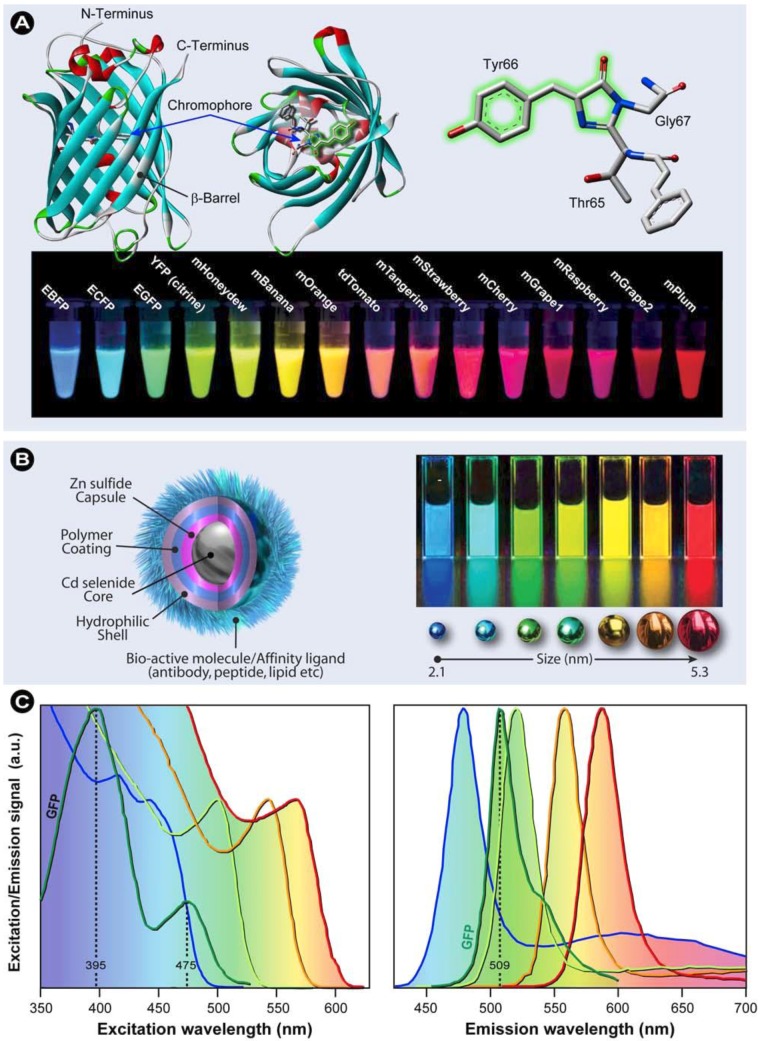
(**A**) Molecular structure and localization of the chromophoric tripeptide in *A. victoria* wild-type GFP. Notice that the tripeptide is located centrally within the β-barrel. A vast number of genetically enhanced (denoted “E”, *e.g.*, EGFP) and engineered FPs [27] have been created over the pasts decades. (**B**) Anatomy of a semiconductor quantum dot (QD), which derives its fluorescent properties from the bandgap between the inner core material and the capsule shell. QDs display size dependent fluorescent properties. (**C**) Excitation and emission spectra of *A. victoria* GFP (green lines) and examples of how the size influences the fluorescent properties of QDs.

The next foremost breakthrough that resulted in an explosion in both instrument and technique development and concurrently biological research was the discovery in the 1960s, sequencing and subsequent development of green fluorescent protein (GFP) as a fluorescent label in the 1990s by Tsien, Chalfie, and Shimomura [22,23,24,25,26,27]. GFP is a 238 amino acid protein that shows bright green fluorescence and was first isolated from the jellyfish *Aequorea victoria*. The 26.9 kDa wild-type GFP consists of a β-barrel structure (Figure 6A) in which the essential chromophoric moiety, an amino acid triplet of Ser65, Tyr66, and Gly67, lies at the centre. It should be noted that the entire poly-peptide structure is necessary for GFP fluorescence−the chromophore forms autocatalytically−and that the molecular structure surrounding the tripeptide influences its fluorescent properties. Furthermore, the protective β-barrel surrounding the tripeptide ensures stability, makes GFP relatively insensitive to environmental influences, and causes physical separation from species such as molecular oxygen. As a result, GFP’s photobleaching rate is lower than those of conventional fluorochromes. Wild-type GFP (from *A. victoria*) fluorescence is characterized by a major excitation peak at 395 nm and a minor one at 475 nm (Figure 6C), which results in bright green emission at 509 nm and a quantum yield of 0.77.

From the moment that the crystal structure was elucidated by Remington’s [28] and Phillips’ [29] groups, researchers have modified GFP through directed and random mutagenesis to, amongst others, expand the color spectrum, to narrow the emission peak, to improve photostability, or to enhance the quantum yield for a particular emission wavelength. Additionally, many fluorescent proteins (FPs) from other species, such as the Anthozoan button polyp *Zoanthus* (ZsYellow), sea anemone *Discosoma* (DsRed), or *Anemonia majano* (AmCyan1), have been identified and isolated, which now results in a wide color palette, with various photostabilities, sensing properties, photo-switchability, and useful FRET pairs (see Table 1 and Table 2).

In recent years, many genetic modifications were made on FPs from various sources, which were denoted according to the corresponding fruit colors, such as mCherry or mBanana, the range of which is exemplified in Figure 6A and a selection of these FPs and their properties are listed in Table 1. The development of FP technology was so significant that it opened the doors to completely new ways of performing fluorescence live cell imaging [23], particularly since it was now possible to tailor the fluorescent label’s properties through genetic engineering and to label proteins by expressing fluorescent fusion constructs directly in living cells. Finally, it should be noted that in the literature FPs are often called “autofluorescent proteins”. Although strictly taken this is correct, such classifications should not be confused with the autofluorescence of endogenous cellular biomolecules discussed previously (*vide supra*).

Additional labeling innovations came from an entirely different discipline of science, which combines nano- and biotechnology. The rapid developments in bionanotechnology over the past decades resulted in the development of luminescent nanoparticles with exceptional physical and chemical properties, not seen in other fluorochromes. Quantum dots (QDs), for instance, are inorganic semiconducting nanoparticles consisting of a core-shell configuration creating a spectral bandgap, *e.g.*, CdSe/ZnS QDs, as depicted schematically in Figure 6B. The size of this bandgap determines the QD’s fluorescent properties and thus the QD’s emission can be directly tuned by their size (Figure 6B) or better said the physical size of the band gap (the band gap energy is inversely proportional to the square of the size of the quantum dot) [30,31,32]. In practice this means that the smaller the QD, the bluer the light. In general, QDs have a relatively long lifetime, which provides the possibility to correct for background signals from short lived fluorescent species by time-gating techniques [33,34]. In addition, it was recently shown that the size of the QD also determines the lifetime, which increases with size [35]. Because QDs have broad absorption spectra, a single light source can be used to excite multiple QDs with different emission wavelengths simultaneously (Figure 6C). This allows the use of both simple voltaic arc lamps and common commercially available lasers, such as argon-ion, helium-cadmium and krypton-argon, with 405 and 488 nm laser-lines, which readily allow excitation of QDs, albeit with varying degrees of efficiency. It is particularly attractive to exploit excitation in the ultraviolet and violet regions (Figure 6C) with blue diode and diode-pumped solid-state lasers that have spectral lines at 375, 405, 442 and 473 nm. Quantum dots are furthermore characterized by a number of additional unique properties [30,31]: (i) QDs are about 10–100 times brighter than organic fluorogenic dyes; (ii) are 100–1,000 times more resistant to photobleaching, because the shell and various coatings form physical barriers that separate the excited state from surrounding biomolecules and molecular oxygen; and (iii) show narrower and more symmetric emission spectra compared with other fluorochromes (typical full-width at half max (FWHM) of ~25–40 nm [36]). A comprehensive description of QD spectral properties is provided by Alivisatos and Biju [30,31,37] and an excellent review on why the small size makes nanoparticles so different from bulk materials is given by Roduner [38].

Nowadays, fluorescence microscopy is the method of choice for live cell imaging and it is a standard procedure to study normal and pathological cell biological processes in single cells, subcellular compartments or across a population of cells by introducing fluorochromes specifically targeted to the (bio)molecules of interest. Major advantages are that fluorescence microscopy techniques provide information with spatio-temporal resolution and are generally less destructive compared with other imaging techniques, *e.g.*, Electron Microscopy (EM).

**Table 1:** Overview of the fluorescent properties of popular organic dyes and fluorescent proteins. Reproduced with permission. © 2011 Carl Zeiss Micro-Imaging GmbH.

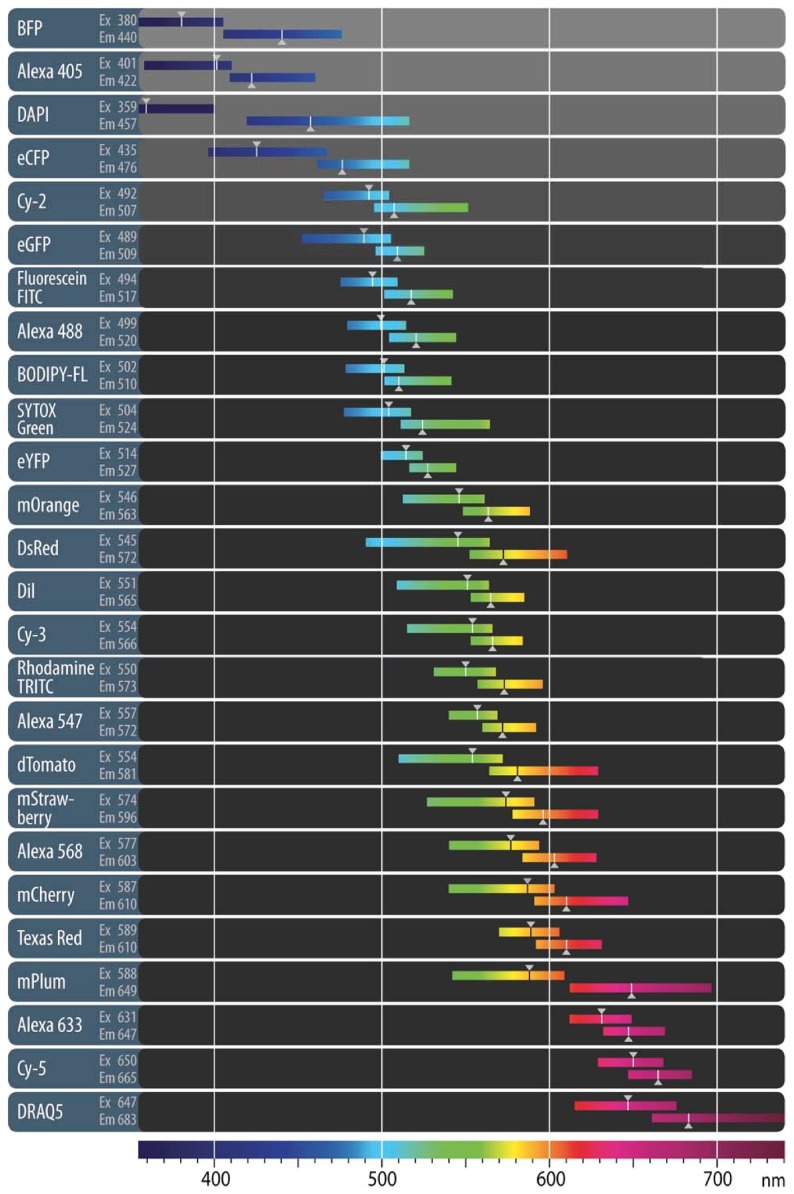

So what makes fluorescence microscopy such a great and specific tool for cellular and molecular imaging and analysis? Essentially it is its selectivity and contrast enhancement. While the selectivity is achieved predominantly through the labeling methods mentioned above, the contrast increase is realized in the microscope itself. Modern fluorescence microscopes can maximize the collection of emitted fluorescent light, while minimizing the collection of the incident excitation light. Thus, one of the main advantages of fluorescence microscopy is the dramatic increase in signal from the labeled structures and molecules against a dark background—analogous to the stars in the black night sky that are not visible during the day. Image contrast critically dependents on the ability of the microscope to pass fluorescent light to the detector (*i.e.*, a CCD camera [charge-coupled device] or a photomultiplier tube [PMT]) while blocking the excitation light. Due to its selectivity and contrast, not only fine cellular and subcellular structures, but even single molecules can be made visible in the fluorescence microscope. If they are spatially well separated and thus not too close to each other or do not light up at the same time, localization of individual molecules is feasible, but is diffraction limited as established by Ernst Abbe (see §1.2.2). 

Three basic components are present in every light microscope, irrespective of the type: (i) an illumination source; (ii) a magnifying lens; and (iii) an image acquisition device. The classical transmitted wide field light microscope typically consists of a white light bulb or light-emitting diode (LED) that illuminates the sample *in toto*, a convex lens system, and the human eye as a detector. Conversely, in a wide field fluorescence microscope, the white light source is replaced by a high power lamp (a mercury or xenon source), which excites the fluorochromes in the fluorescently labeled sample and induces fluorescence emittance as shown schematically in Figure 5. Images are typically acquired visually by eye or electronically with a CCD camera. As described above, fluorochromes have characteristic excitation spectra. Thus, an appropriate excitation filter, usually a band-pass filter (BP), is placed between the lamp and the sample to narrow the wavelength range of light reaching the sample to such an extent that the fluorochromes used are excited efficiently, whereas unwanted excitation is minimized. Since the emitted light has a longer wavelength than the excitation light, an emission filter (either a long pass [LP] or BP filter) placed between the sample and the detector effectively blocks the excitation light and prevents perturbation of the final image. Intense light is required for successful fluorescence excitation. Lasers generally produce high intensity light and have proven to be an excellent alternative excitation source to mercury and xenon lamps. Accordingly, lasers are now commonly utilized in confocal and multiphoton laser scanning microscopy to point illuminate the sample. Since lasers are a source of monochromatic light, generally no excitation filter is required. However an emission filter or an alternative spectral selection device is still needed to stop the excitatory laser light from reaching the detector and to tune in on the fluorochrome’s emission signal, especially in multiple labeling experiments. In the case of a confocal laser scanning microscope, the mercury lamp is replaced by a set of appropriate lasers that allow the excitation of various fluorochromes, the excitation filters are removed, and the detecting camera is replaced by a photomultiplier tube [39,40,41]. Lichtman and Conchello recently published a good and concise introduction into fluorescence microscopy [42], whilst more extensive reviews can be found in Pawley’s handbook [39].

#### 1.2.2. Resolution in Fluorescence Microscopy

**Figure 7:**
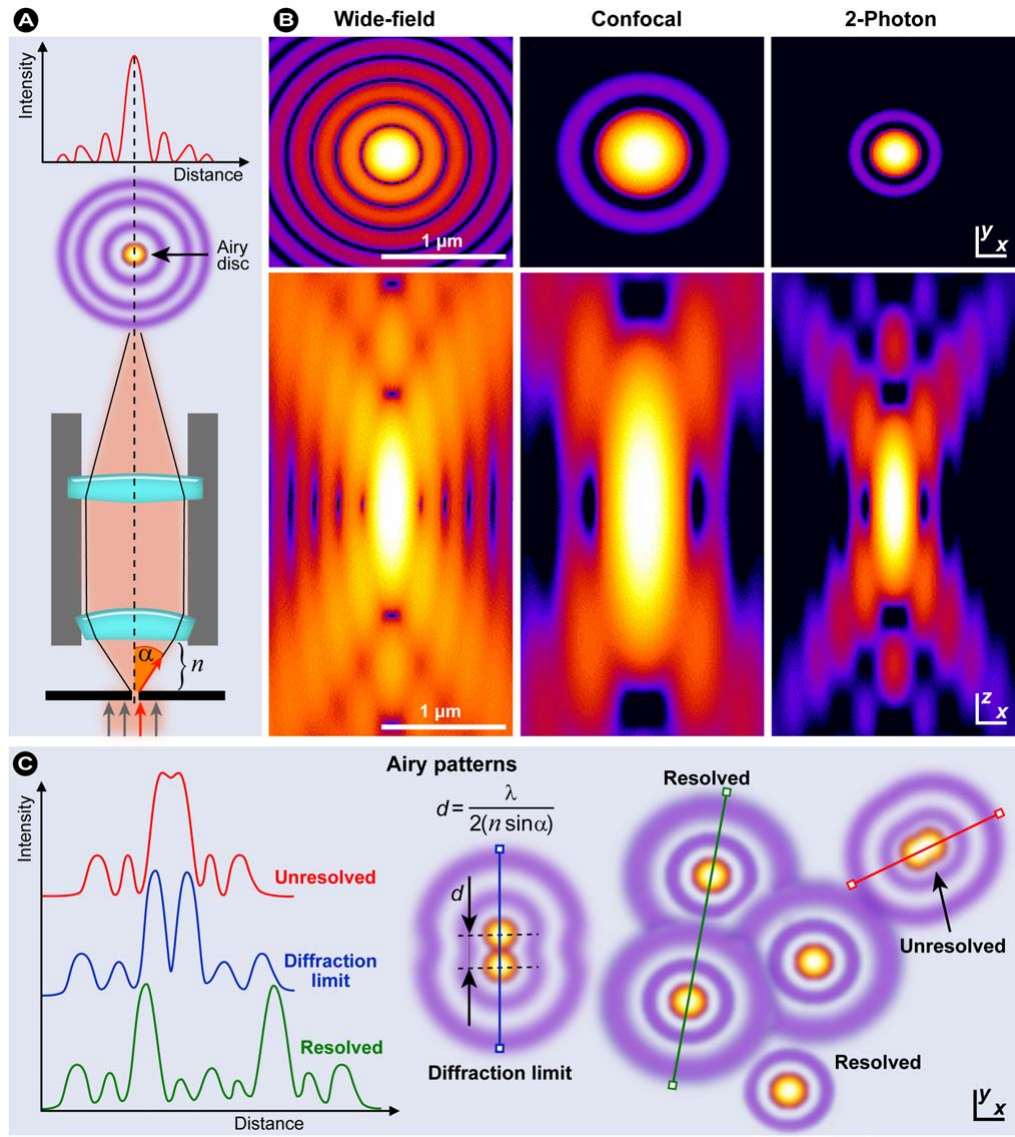
(**A**) Objective and light beam path: a point source in the focal plane is imaged and projected as a bright central spot (Airy disc) and a concentric ring pattern (Airy pattern) that results from diffraction. (**B**) Calculated x-y (above) and x-z (below) intensity distributions (logarithmic scale) for a point source imaged with various microscopical techniques. Optical conditions: λ_ex_ = 488 nm and 900 nm for 1PE and 2PE, respectively; λ_em_ = 520 nm; N.A. = 1.3 for an oil immersion objective with oil refractive index value set at 1.515. Reproduced from [45]. © 2006 BioMed Central. (**C**) Schematic diagram of an Airy disc diffraction pattern: Abbe diffraction limit, contrast, and optical resolution.

In fluorescence microscopy, the optical resolving power determines the amount of detail observed in a specimen, which in turn is determined by a number of physical factors and instrument limitations. If light hits a small object under observation, the direction of the incidence light is changed (diffraction) and this deflection increases with decreasing size of the object. In order to obtain sharp images, the objective must capture as much of the deflected light as possible, which is achieved with wide angular openings (aperture). Ernst Abbe first defined the numerical aperture (N.A.) [43], which determines the objective’s light capturing capacity and can mathematically be written as:


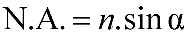
 (5)

where *n* is the refraction index of the medium between the object and the objective (*n* = 1 for air and for immersion oil *n* = 1.51), and α is half the objective opening angle (Figure 7A). To maximize capturing the deflected light and to increase the resolution, several strategies can be employed, including the use of a condenser lens for illumination to widen the angle of the ray cone on the illumination side and/or to use an immersion liquid between objective lens and the cover slip, which abrogates reflections that normally diminish the resolving power. In air, theoretically N.A. = 1 can be achieved when α = 90°, but in practice, N.A. values above 0.95 cannot be attained. Conversely, with immersion oil, N.A. values larger than 1 can easily be reached and thus the use of oil immersion objectives is the only way to increase magnification with sufficient resolution and contrast. 

So what exactly is resolution or optical resolving power? The introduction of several parameters and concepts are required to delineate the concept of resolution. First of all, it is important to realize that in fluorescence microscopy the resolution is not directly governed by the magnification. Secondly, resolution and contrast are two reciprocally interrelated parameters that are important in fluorescence microscopy and should not be considered as separate entities. Intuitively it becomes clear that when the contrast approaches zero, it would be futile to discuss optical resolution. As stated previously, the individual objects in a specimen cause diffraction of light and as a result, an illuminated point source within the specimen is observed as a bright central spot (Airy disc) with surrounding diffraction rings (Airy pattern) as shown in Figure 7. It was George Biddell Airy who first theoretically described this phenomenon [44] in 1835, although others had previously observed it experimentally. Generally, optical microscopes can be assumed to be linear and shift-invariant, which means that the image of a specimen essentially consists of the linear superposition of all the specimen’s individual elements, which results in the final image, *e.g.*, a cell with all its labeled components and organelles. The intensity point spread function (PSF) characterizes such a system and the Airy pattern is essentially the intensity distribution of the intensity PSF in the focal plane (x–y). Combining both the PSF in lateral (x–y) and axial (x–z) direction (see Figure 7B) creates a complex three dimensional shape, the three dimensional PSF, which characterizes the response of the entire optical system, lenses, mirrors, optical apertures, and imperfections or misalignments in the optical system to the illuminated point source and the diffraction those elements and the object cause. 

Essentially, resolution may be defined as the smallest distance between two points in the specimen that can still be discriminated as separate points (Figure 7C) *at a particular contrast*, which with Equation 5 can be written as:


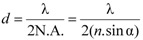
 (6)

**Figure 8:**
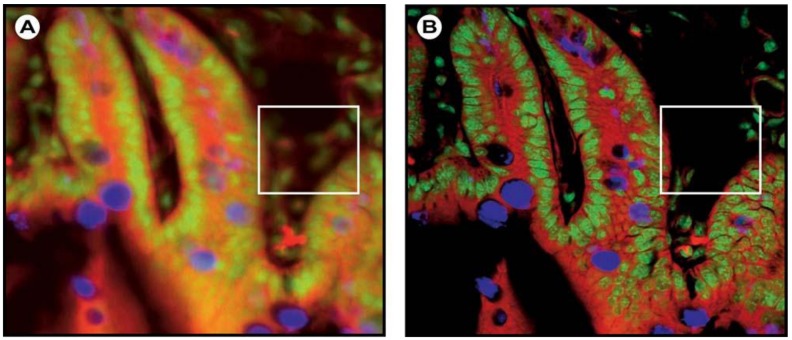
Confocal versus wide field microscopy. Wide field (**A**) and confocal (**B**) image of a triple-labeled cell aggregate (mouse intestine section). In the wide field image, specimen planes outside the focal plane degrade the information of interest from the focal plane, and differently stained specimen details appear in mixed color. In the confocal image (**B**), specimen details blurred in wide field imaging become distinctly visible, and the image throughout is greatly improved in contrast. Notice that out of focus signals in the wide field image cause additional structures to appear (white box). Reproduced with permission. © 2011 Carl Zeiss Micro-Imaging GmbH.

Contrast is defined as the difference between the maximum intensity and minimum intensity occurring in the space between two objects of equal intensity (the Airy discs; contrast = 1) [46,47]. When the two point objects are well separated, the contrast minimum between them is near zero and the objects can be discriminated (they are resolved) as shown in Figure 7C (green). However, as the point objects approach and their PSFs start to overlap, the intensity minimum between the two object maxima is reduced until the objects are no longer resolved (Figure 7C, red). Even though this formulation closely follows practical microscopy, commonly the Rayleigh criterion for resolution is used, which states that two points are resolved when the first minimum of one Airy disc is aligned with the central maximum of the second Airy disc. From the above mentioned considerations, it becomes obvious that the smaller the Airy pattern (Figure 7B), the higher the resolution, the more detail can be obtained in an image (compare wide field and confocal microscopy in Figure 8).

It was Ernst Abbe who in 1873 theoretically laid the foundation for describing the diffraction limit that led to the formulation of Equation 6. He described that the smallest resolvable distance between two points using a conventional light microscope cannot be smaller than half the wavelength of the imaging light [48]. Consequently, an increase in resolution can only be achieved if the wavelength of light used is as small as possible. Thus if a wavelength of 400 nm is used, which is still in a range that is usable in biological imaging, an approximate lateral resolution of 200 nm is feasible. However, in comparison to the size of a single animal cell (10–100 µm), viruses (20–200 nm), and organelles (500 nm–10 µm), this still precludes attaining sufficient resolution. Further reduction of the wavelength into the UV causes significant damage to biomolecules and in live cell imaging seriously affects and alters normal cellular function and homeostasis. Consequently, the measured results are perturbed and evaluation of the biological function cannot be performed with sufficient accuracy and thus the results might be scientifically flawed.

#### 1.2.3. Confocal Laser Scanning Microscopy (CLSM)

**Figure 9:**
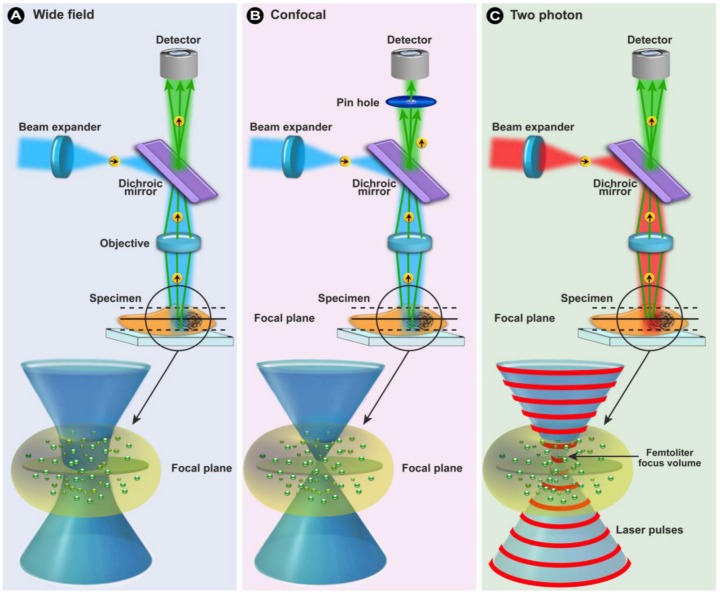
In confocal microscopy (**B**) the concept of illuminating the sample with excitation light (*e.g.*, blue light) and the sample emitting light with a longer wavelength (*e.g.*, green light) is identical to the general principles of fluorescence in wide field microscopy (**A**). The differences to wide field microscopy are: (i) the excitation laser light is scanned over the sample and the emitted light originates from this area; (ii) on the detection beam path a pinhole aperture in front of the detector prevents light emitted from above or below the focal plane (dotted lines) from reaching the detector; and (iii) because only light from the focal plane (solid line) reaches the detector an optical section is generated. (**C**) In two-photon microscopy, a high flux of excitation photons from a pulsed laser caused the simultaneous absorption of 2 long wavelength photons and emission of a photon with a shorter wavelength (anti-Stokes). Because excitation is restricted to a small femtoliter focal volume, out-of-focus emission is negligible and thus no pinhole is required.

Confocal laser scanning microscopy (CLSM) is a technique, which combines high-resolution optical imaging with depth selectivity [39]. The original technique used a stage-scanning confocal optical system and was invented by Marvin Minsky in 1957 [49]. Essentially, the CLSM is based on a conventional optical microscope in which instead of a lamp, a laser beam is focused onto the sample and an image is built up pixel-by-pixel by collecting the emitted photons, usually with a PMT. Thus, CLSM combines point-by-point illumination with simultaneous point-by-point detection (Figure 9) and illumination and detection are restricted to a single diffraction-limited point. A key feature of CLSM is its ability to acquire well focused images from various depths within the sample; a process called “optical sectioning”. The images of a mouse intestine section in Figure 8, noticeably illustrate the gain in resolution in CLSM imaging over conventional wide field imaging.

This is achieved by placing a small pinhole aperture before the detector (Figure 9B), which prevents emitted out-of-focus light from the planes above and under the focal plane, as well as stray light from reaching the detector.

In conventional wide field microscopy, such signals cause glare, distortion and blurriness in the image and these artifacts are collectively called “convolution”. Even though a small improvement in both axial and lateral resolution (notice the central maxima in Figure 7B) is achieved over wide field techniques, the abrogation of interference of out-of-focus and stray light with the in-focus signal and a significant reduction in the diffraction pattern (Figure 7B) cause a considerable increase in resolving power. The focal point in the sample and the pinhole lie in conjugate planes, as shown in Figure 9B, and this optical arrangement of the focal points is called ‘confocal’. Notice the difference in the light pathways over a wide field fluorescence microscope in Figure 9. The smaller the pinhole, the less light from out-of-focus areas within the specimen reaches the detector, the lower the intensity of the image. During scanning, with a defined focusing along the z-axis (axial direction) and lateral movement (x and y axis), the confocal volume element is moved through the specimen by a succession of object planes. It is thus possible to obtain optical sections of the specimen and reconstruct its 3D structure. 

In biological imaging applications, may that be single cells, tissues, or intact model organisms, CLSM has proven its power over the past thirty years and concomitantly produced spectacular new scientific insights, particularly because of its optical sectioning capability and the possibility for 3D reconstruction from a stack of individual sections. Furthermore, the advantage of this technique comes from its non-linear behavior in that the technique is sensitive to the square of the light intensity and not just the light intensity. This combined with the increase in contrast by cutting off unwanted signals from out-of-focus planes, which results in sharper images with better z-resolution (Figure 8), clearly offers an advantage over the wide field microscope [50,51].

#### 1.2.4. Multiphoton Fluorescence Microscopy

Multiphoton fluorescence microscopy resembles CLSM in that both use focused laser beams to scan a specimen in a raster pattern (point-by-point) to generate images, and both have an optical sectioning capability. Unlike confocal microscopes where the optical sectioning capability is generated in the emission light path, in a multiphoton microscope the sectioning capabilities are caused on the excitation side because of the extraordinary way that the fluorochromes are excited. The concept of multiple photon excitation (MPE) was first theoretically described by Maria Göppert-Mayer in 1931 in her doctoral thesis [52] and subsequently observed experimentally in 1961 by Kaiser and Garret when they detected blue fluorescence in CaF_2_ : Eu^2+^ crystals in response to a red 0.5 ms pulsed ruby laser [53]. However, it was Winfried Denk who developed two photon imaging for use in living cells and tissues in the lab of Watt Webb [54].

**Figure 10:**
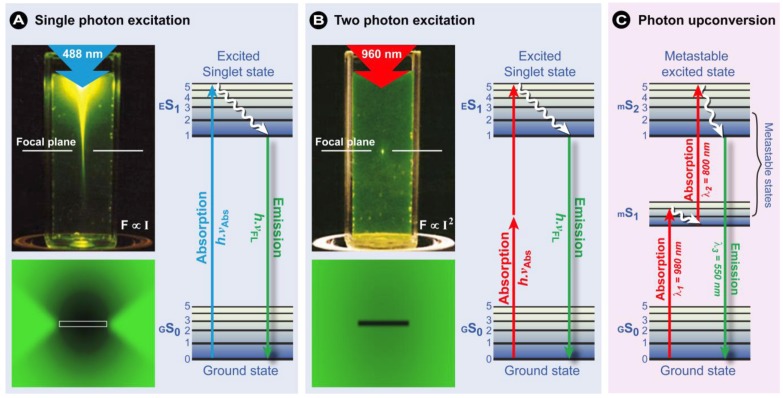
Comparison of the excitation profiles of (**A**) single photon, (**B**) two photon, and (**C**) upconverting excitation. From the light excitation pattern with 488 and 960 nm (0.16 NA) lasers in the cuvettes, it can clearly be seen that only in two photon excitation (**B**), the excitory beam is focused in a spot in the focal plane. Conversely, in single photon excitation (**A**), additional light emanates from above and below the focal plane. Bottom figures: repetitive scanning in the focal plane (x-y plane) in a fluorescein-stained formvar film shows that in two photon excitation only the focal plane photobleaches. The Jabłoński diagrams illustrate the differences in photon absorption between the various systems. In TPE, two photons of the same wavelength must arrive simultaneously in time and space to excite the electron. Conversely, upconverting fluorochromes **(C)** contain metastable states, as in this example for Europium (III) ions, which are sufficiently stable to allow sequential absorption of long wavelength photons. As a result, both TPE and photon upconversion show anti-Stokes shifts. Partially reproduced from [59] with permission from Macmillan Publishers Ltd: Nature Biotechnology © 2002.

Multi-/two-photon excitation (TPE) is based on the simultaneous absorption of two (or multi) photons by the fluorochromes in a sub-femtoliter volume at the focus (Figure 9C) in which the energy for exciting the electron from the ground state to the excited state is provided by two photons with approximately half the energy (Figure 10B). Since energy scales inversely with the wavelength according to Planck’s law (Equation 1), half the energy means twice the wavelength of an excitory photon in single photon excitation (compare Figure 10A,B). Since the statistical probability of the concomitant absorption of photons is extremely low, high local photon fluxes are required (MW/cm^2^ to GW/cm^2^ [55]), which only became possible with the introduction of femtosecond mode-locked pulsed lasers. Two-photon excitation is a non-linear process, as the absorption rate increases with the second power of the excitation light intensity. As a consequence, even when using high power pulsed lasers, the excitation is restricted to a small volume in the focal plane of the specimen (see schematically in Figure 9C and in the fluorescein solution in Figure 10), where the photon density is high enough. The practical outcome of this phenomenon is an optical sectioning without the need for a pinhole to block fluorescence from out-of-focus locations (Figure 9C). By subsequently scanning this excitation volume through a sample, z-stacks of 2-dimensional images can be collected and 3-dimensional images can be reconstructed.

An additional benefit of limiting the excitation of the fluorochromes to such a small volume in the plane of focus is a significant reduction in the overall photobleaching. This is exemplified in Figure 10 when a fluorescein-stained formvar film is illuminated in either a CLSM or a TPE mode. The bleaching pattern in the film clearly shows significant bleaching above and below the focal plane in CLSM, whereas bleaching is exclusively restricted to the focal plane in TPE microscopy. Overall, this significantly reduces the photodamage and cytotoxicity normally associated with fluorescence microscopic imaging experiments and ensures that near-normal cellular homeostasis is maintained. Consequently, cells may be observed for longer periods of time with fewer toxic effects. Another important advantage of this technology is that, since most fluorochromes are excited in the range from 350–550 nm, deep red or infra-red excitation light can be used in TPE, which penetrates much deeper into the specimen due to reduced scattering and absorption by endogenous chromophores. A variant, two-photon autofluorescence microscopy (2PAM), exploits the autofluorescence of endogenous biomolecules to assess the disease states in tissues based on changes in morphological, spectral, and lifetime parameters. Since, for instance cell layer thickness can be used as an indicator of dysplasia and carcinoma, the imaging depth capabilities of TPE are particularly important in 2PAM. Durr *et al.* determined that *ex-vivo* 2PAM imaging in a human tongue biopsy was feasible to a depth of 370 μm [56]. In normal laser scanning devices, only a penetration depth of about 250 μm can be reached. Some researchers have used high-pulse power regenerative amplifier systems [57,58] to increase imaging depth and Denk *et al.* reported imaging at a depth of 1,000 µm in living mouse brains by use of a Ti:Al_2_O_3_ regenerative amplifier [58].

The increased achievable imaging depth is a significant advantage of TPE microscopy over conventional CLSM methods. Other non-linear methods, such as second harmonic-based microscopy or the use of fluorochromes that show upconverting properties have also been developed for a myriad of biological applications. What all these methods have in common is that their excitation and emission follow anti-Stokes shifts, *i.e.*, the excitation wavelength is larger than the emission wavelength.

Second-harmonic imaging microscopy (SHIM) is based on the nonlinear optical effect of second-harmonic generation (SHG) in which laser light is focused on a sample to generate frequency-doubled light (two photons with wavelength λ are extinguished to generate a single photon with wavelength 0.5λ [60,61]). Unlike in TPE, SHG does not involve an excited state and therefore no energy is lost during relaxation of the excited state as in the case of TPE. Furthermore, SHG and SHIM offer several advantages in live cell imaging: (i) SHG is energy conserving; (ii) conserves laser coherence; (iii) requires no labeling; (iv) since no excited state is involved, photobleaching and phototoxicity are virtually absent; and finally (v) has sectioning capabilities because in SHG the amplitude is proportional to the square of the incidence light intensity as in TPE [60,61].

In contrast to TPE, in photon upconversion, the chromophore’s properties allow the sequential absorption of long wavelength photons. The fundamental processes involved in upconversion are complex and consist of several competing processes, including sequential energy transfer, excited state absorption, and phonon interaction (a quantum of vibrational energy that arises from oscillations within a crystal) [62,63,64]. Generally, rare earth metals (d- and f-block elements) such as lanthanides are involved as luminescent complexes with organic enhancers, or as dopants in luminescent nanoparticles. Less effective upconversion is achieved when using actinides or transition metal ions [62,63]. Materials with upconverting properties are often referred to as “upconverting phosphors”, which is potentially a confusing term. As illustrated in Figure 10C, the occurrence of metastable excited states with long lifetimes in the ms range abolish the need for simultaneous arrival of the photons and thus high local photon fluxes. For upconverting chromophores, a normal fluorescence microscopic setup with low power lasers–intense anti-Stokes emission is already possible below 1 W/cm^2^ [64,65]–inexpensive detectors, such as a PMT for photon counting, a standard long-pass excitation filter, and a narrow band-pass emission filter with adequate infrared blocking capabilities generally suffices as instrumentation. The extraordinary photoluminescent characteristics in upconverting materials allow: (i) imaging against dark backgrounds, since upconversion does not occur in endogenous cellular biomolecules; (ii) with N(IR) excitation, excellent imaging depths can be achieved; (iii) the use of conventional imaging systems keeps investments and costs low, (iv) their photoluminescent properties are relatively insensitive to environmental changes, such as pH or solvent polarizability; (v) virtually no photobleaching occurs, and (vi) such materials have long shelf-lives. More in-depth deliberations on upconversion can be found in references [13,62,63,64,66].

From the above considerations it quickly becomes clear that multiphoton fluorescence microscopy is a powerful tool in biomedical research that offers reduced photobleaching and low photo-toxicity, higher penetration depths, and higher spatial resolution than other *in vivo* imaging modalities. With the continuous development of new fluorescent proteins, equally multiphoton microscopy is subject to constant improvement. Furthermore, it can be deduced from the literature that in life science research, predominantly two-photon techniques are now commonly applied to resolve scientific questions. A comprehensive guide to choosing the right fluorescent protein and excitation wavelength for two-photon applications and a review on the two-photon spectral properties of fluorescent proteins was recently presented by Drobizhev *et al.* [67]. Excellent reviews on two and multiphoton microscopy, including a deliberation of the advantages and disadvantages may be found in [45,47,54,55,59,68,69,70,71].

## 2. Photobleaching-based Techniques for Assessing Cellular Dynamics

### 2.1. Fluorescence Recovery after Photobleaching (FRAP)

FRAP was developed in the 1970s by Axelrod and coworkers as a technique to study protein mobility in living cells by measuring the rate of fluorescence recovery at a previously bleached site [72,73]. Originally the FRAP technique was utilized as a method to measure diffusion in cellular membranes [74,75] by using organic dyes such as fluorescein. However, with the development of both fluorescent protein technology and confocal microscopy, FRAP became popular for studying protein mobility in the cell interior. A major benefit of genetically tagging proteins is the fact that now studies on living cells were possible devoid of disruption of the cell and cellular homeostasis by micro-injection or permeabilization techniques. Furthermore, both screening and genetic engineering increased the number of fluorescent proteins to such an extent that they virtually cover large parts of the spectrum, allowing imaging with multiple labels, as stated previously. As a result, the scope of FRAP, from those early studies on diffusion of plasma membrane proteins and phospolipids [76,77,78,79,80] expanded not only to address diffusion rates, but protein dynamics and interactions with other cellular components [81,82,83,84,85]. Most importantly, FRAP has been shown to be a good approach to study nuclear protein dynamics in living cells and was further developed by researchers such as Adriaan Houtsmuller [54,92].

Because FRAP is such a versatile method, it has become a common technique for studying dynamics in almost all aspects of cell biology, including cytoskeletal dynamics [86,87,88], vesicle transport [89,90,91,92], cell adhesion [93,94], mitosis [95,96,97], chromatin structure [98,99,100,101], transcription [98,99,102], mRNA mobility and DNA-interacting molecules [103,104,105], protein recycling [106,107,108] and signal transduction [109,110] to name but a few.

Recapitulating, FRAP is generally suitable to study and investigate:

➲ Protein/molecule movement and diffusion (diffusional speed).
➲ Compartmentalization and connections between intracellular compartments.
➲ The speed of protein/molecule exchange between compartments (exchange speed).
➲ Binding characteristics between proteins. Additionally, the effect of mutations that alter individual amino acids on protein association, and the effect of small molecules, such as drugs or inhibitors, on protein pairs can effectively be studies using FRAP.
➲ Immobilization of proteins that bind to large structures, *e.g.*, DNA, nuclear envelope, membranes, cytoskeletal elements, *etc*.

#### 2.1.1. The Basic Principles of FRAP

**Figure 11:**
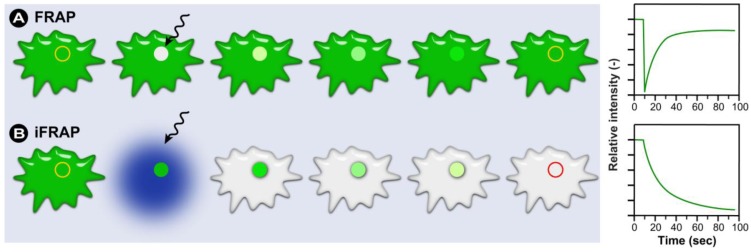
Schematic representation of a FRAP and iFRAP experiment. (**A**) A region of interest (ROI) is selected, bleached with an intense laser beam, and the fluorescence recovery in the ROI is measured over time. (**B**) In iFRAP, the reverse is done and a ROI is selected to remain intact, while the rest of the cell is bleached. This is particularly useful when studying dynamic movement in organelles such as the nucleus.

In a typical FRAP experiment (Figure 11A), fluorescent molecules are irreversibly photobleached in a small area of the cell by high intensity illumination with a focused laser beam. Subsequently, diffusion of the surrounding non-bleached fluorescent molecules into the bleached area leads to recovery of fluorescence with a particular velocity, which is recorded at low laser power.

As previously described, photobleaching in most fluorochromes requires excitation to an excited state and the presence of molecular oxygen. During FRAP, the high light intensity in the presence of molecular oxygen causes irreversible damage to the fluorochrome (Figure 4), thereby permanently interrupting the cycle of repetitive excitation and photon emission. Ultimately, those fluorochrome molecules that are permanently damaged no longer contribute to the recovery of fluorescence in the bleached area. The bleached fluorochromes are replaced by unbleached ones, a process that occurs as the result of the diffusional exchange between them. 

The fraction of fluorescent molecules that can participate in this exchange is referred to as the mobile fraction (*M_f_*), whereas the fraction that cannot exchange between bleached and non-bleached regions is called the immobile fraction (*I_f_*), as shown in Figure 13A. Therefore, FRAP provides important insights into the properties and interactions of molecules within the cellular environment. 

**Figure 12:**
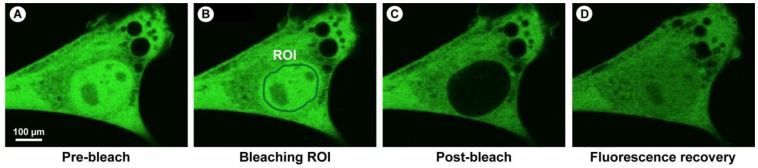
Example of a FRAP experiment to show that monomeric GFP can pass the nuclear membrane. (**A**) Myoblast cell line (myo3) homogenously expressing GFP-Myosin III before bleaching. (**B**) A region of interest (ROI) is bleached with high intensity laser light. Directly after bleaching, the cell shows a dark area in which the fluorochromes were permanently damaged and thus no longer emit light (**C**). The fluorescence in the photobleached region recovers via replacement with intact fluorochrome molecules from the surrounding area (**D**). Note that the total amount of fluorescence has decreased during the experiment, because a substantial amount of fluorochromes were irreversibly damage.

FRAP can also be used to measure the dynamics of 2D or 3D molecular mobility, *e.g.*, in diffusion, transport, or any other kind of movement of fluorescently labeled molecules in living cells. A representative example is given in Figure 12, which shows that monomeric GFP-Myosin III can easily traverse the nuclear envelope membrane. Myosins are motor proteins that together with kinesins and dyneins are responsible for a wide range of movement and transport processes. Class III myosins play critical roles in the vertebrate retina and inner ear function and show an exceptionally high affinity for actin [111]. The nucleus is bleached with high intensity (~500 ms; >30 mW) with a 488 nm laser. Subsequently, the nucleus is devoid of green fluorescence. Over time the fluorescence recovers and reaches a plateau. Notice by comparing Figure 12A,D that the total fluorescence intensity decreases, because a significant number of fluorochromes were irreversibly bleached. 

**Figure 13:**
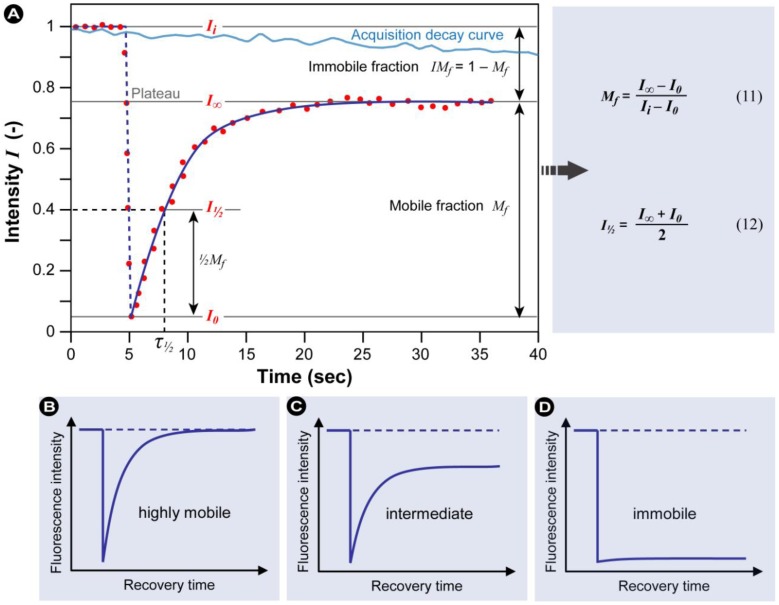
Anatomy of a typical FRAP curve. (**A**) From the initial (pre-bleach) fluorescence intensity (*I_i_*), the signal drops to a particular low value (*I_0_*) as the high intensity laser beam bleaches fluorochromes in the ROI. Over time the signal recovers from the post-bleach intensity (*I_0_*) to a maximal plateau value (*I_∞_*). From this plot and equations 11–12, the mobile fraction (*M_f_*), immobile fraction (*IM_f_*), *I*_½_ and corresponding time (*τ*_½_ – the time for the exchange of half the mobile fraction between bleached and unbleached areas) can be calculated (Light blue line: reference photobleaching curve to correct for fluorescence loss during data acquisition). The information from the recovery curve (from *I_0_* to *I_∞_*) can be used to determine the diffusion constant and the binding dynamics of fluorescently labeled proteins. Based on different recovery profiles, the protein mobility can be classified as (**B**) highly mobile with virtually no immobile fraction, (**C**) intermediate mobile with an immobile fraction, or (**D**) immobile.

In FRAP experiments, the images are analyzed and processed to generate a kinetic plot of photobleaching by displaying the temporal fluorescence changes in the bleached region of the cell. From this plot, the mobile and immobile fractions can be determined by calculating the ratios of the final to the initial fluorescence intensity (see Figure 13 and the equations therein). By convention, the speed of recovery to half the plateau intensity (I_∞_) is called ‘half maximum’ or ‘half life’ (τ_½_). The shorter the half life, the faster the fluorescence recovery occurred and the higher the diffusion. Furthermore, the half life of recovery is proportional to the bleach area size if the recovery is diffusion limited.

A more absolute way of obtaining the half life and immobile/mobile fractions, which is also suitable for automation, is through non-linear curve fitting of the experimental data points using a simple exponential equation:


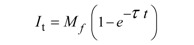
 (7)

Subsequently, the fitted coefficients can be used to extract the required information from the FRAP curve with: 

Mobile fraction*= M_f_* (8)

Immobile fraction (*IM_f_* ) = 1 – *M_f_* (9)

Substitution of *I_t_* with ½ *M_f_* results in an expression for the half life:


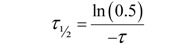
 (10)

From Equation 10, the half life can be calculated from τ, which provides information on the diffusion of the fluorochrome. This is just a concise and simple example on how to extract information from FRAP curves via modeling. Many methods are currently available to analyze FRAP data curves ranging from biochemical binding models to molecular transport modeling and are often included in the software provided by the microscope manufacturer. An in-depth overview of analytical methods, including potential pitfalls in data analysis is provided in references [102,112,113,114,115,116,117,118,119].

Different profiles of the temporal fluorescence recovery intensity plot provide information about the protein’s mobility, which can be classified as high, intermediate or immobile (Figure 13B–D).

Changes in the mobile fraction may also give clues about various intracellular processes and their temporal outcomes, *e.g.*, interaction of a protein of interest with, for instance, other proteins or (bio)molecules. The mobile fraction can also be markedly affected by cellular membrane barriers and micro-domains within the membrane. These discontinuities can prevent, or temporarily restrict, the free diffusion of molecules through various cellular compartments or within the membrane itself. Conversely, active transport via coated vesicles or motor proteins, such as the aforementioned myosins (Figure 12) that use actin filaments to ATP-dependently transport cargo over large distances, can cause a significantly higher mobility compared with diffusion limited processes. Such information can easily be extracted from FRAP data curves.

#### 2.1.2. Practical Aspects and CLSM-Specific Considerations

In any FRAP experiment, a series of fluorescence images is initially collected to give a baseline value for the intensity in both the ROI and the surrounding labeled cellular environment. Following this, a defined region of the sample is illuminated with high intensity light causing photobleaching of the fluorochromes within this region. This creates a darker, bleached region within the sample. Photobleached molecules are subsequently replaced by non-bleached molecules over time, leading to an increase in fluorescence intensity in the bleached region, as described previously (Figure 11A and Figure 12).

The intensity of the scanning laser applied during the acquisition of typical confocal images is often sufficient to produce significant bleaching of the entire sample within the field of view during the course of image acquisition. Each point in the scanned sample receives the same total light intensity, resulting in uniform bleaching of the whole sample. One way of correcting for this acquisition bleaching is through mathematical corrections (see Equation 13). Additionally, a number of fundamental and instrumental approaches can be employed to minimize this background bleaching *ab initio*. For example, applying line scans instead of 2D scans, using fluorescent probes that are less susceptible to photobleaching, decreasing the laser power or the pixel resolution by zooming out or using faster scans. In these cases, a compromise between temporal and spatial resolution in a time course experiment needs to be take into consideration. For example, if images are acquired at high speed, the spatial information is often lost, but if the images are acquired slowly in order to increase spatial resolution or gain a better signal-to-noise ratio, the information about the dynamic processes occurring in the biological sample may be lost. Consequently, in quantitative FRAP experiments the crispness of the image itself is often less important and therefore the spatial resolution is sacrificed for the benefit of maximizing the temporal resolution. In practice, the FRAP data (fluorescence intensity values) are acquired quickly in order to successfully record the recovery of the bleached region. However, this results in a significant decrease in the signal-to-noise ratio, which can be partly compensated for by increasing the aperture of the confocal pinhole.

Although undesirable, a small degree of background bleaching can be tolerated and corrected for. Because background bleaching is effectively constant throughout the field of view, the fluorescence intensities in a region of the cell some distance from the bleached region, or in another cell in the field of view, can be used to correct the images for this effect. In practice, curve fitting is performed with an exponential equation that contains an extra term to correct for the bleaching that occurs during image acquisition. Many methods can be employed, ranging from recording an image acquisition decay curve (light blue curve in Figure 13A) and correcting the FRAP curve in every single time point [120] to expansion of equation 7 with a term that assumes that the acquisition bleaching follows a simple exponential decay, such as used in the “back multiplication method”. The normalized, uncorrected FRAP curve is fitted according to:


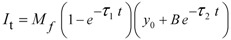
 (13)

The values for τ_2_, y_0_, which is the minimum plateau that the acquisition decay curve approaches as t→∞, and *B* are obtained from fitting the decay curve recorded either by measuring the decay in the whole cell, an adjacent cell (both whole cell ROI), or a reference region (reference ROI). Furthermore, a myriad of modeling methods based on the kinetics of the molecular phenomenon are available, as described in references [102,112,113,114,115,116,117,118,119].

The ideal fluorescent probe for use in photobleaching studies should be highly fluorescent (high quantum yield), but only moderately susceptible to photobleaching. This permits bleaching within realistic time frames, but limits bleaching during image acquisition. Green fluorescent protein is the most widely used fluorescent probe for cellular studies because of its stability, low cytotoxicity, because it does not bleach significantly at low light intensities, does not seem to be damaging to the cell after undergoing irreversible photobleaching (see § 1.2), it can be readily expressed in several cell types where it is fused to a particular protein, and in many cases tagging a protein of interest with GFP has no significant influence on the function and localization of the protein under investigation [85,121,122,123,124]. Because of these characteristics, GFP behaves more like a “non-invasive” extrinsic fluorochrome, with a stability that is higher than the commonly used organic dye fluorescein isothiocyanate (FITC) [125]. FITC is neither the ideal probe nor a good choice for performing quantitative FRAP-studies with the CLSM, exactly because of its high susceptibility to background bleaching during image acquisition. A good alternative for use in FRAP studies are the Alexa series of dyes, because of their superior properties, such as their high fluorescence quantum yields and relatively low propensity to bleaching during image acquisition, but still allow efficient bleaching in the ROI with an adequate high dose of laser light [126]. 

#### 2.1.3. Inverse FRAP (iFRAP) in Cell Biology

A modified version of the FRAP method, called inverse FRAP (iFRAP) was initially developed to study the mobility of molecules in small areas of the nucleus and their exchange with the surrounding nucleoplasm [98,127]. Inverse FRAP, as schematically depicted in Figure 11B, was initially developed by Misteli *et al.* [98] and from its setup is particularly useful to study the residency time of molecules in small organelles. In iFRAP, the entire population of fluorochromes in the cell is bleached, except the accumulated fluorochromes in a small part of the organelle. Subsequently, the loss in fluorescence in the accumulation is recorded over time (Figure 11B), from which the rate of exchange with the surroundings can be calculated. Since this loss in intensity directly reflects the releasing process between molecules, no further complicated analytical methods are required to determine the rate of exchange [122]. One of the main limitations of iFRAP lies in the long time needed to photobleach the entire cell (this can take several seconds), which renders iFRAP unsuitable to detect fast translocations. Therefore, iFRAP is mostly useful for analyzing the dissociation kinetics of molecules bound to an immobile intracellular structure.

**Figure 14:**
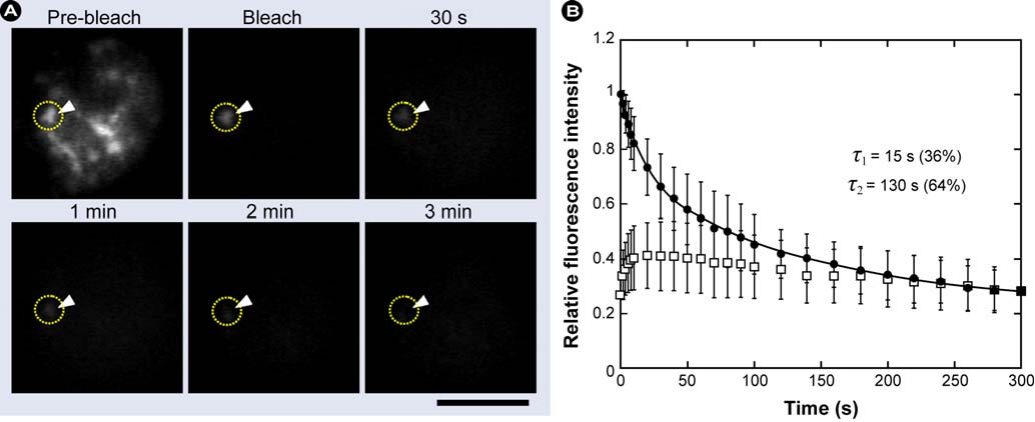
iFRAP experiment showing the dissociation of pre-mRNA from speckles. (**A**) Fluorescence images of a Cos7 cell micro-injected with pre-mRNAs before and after photobleaching of the nucleus except for the speckles region (ROI) shown by the arrowheads and circle. Scale bar, 10 μm. (**B**) The changes in fluorescence intensity after photobleaching at time 0 at the speckles (closed circles) and in its adjacent photobleached nucleoplasm (open squares) was plotted as a function of time. The curve shows a rapid dissociation, followed by slow-dissociation from speckles and slightly increased fluorescence intensity at the adjacent nucleoplasm for about 10s after the photobleaching and then reaching a value similar to that of the speckles. Reprinted from [127] with permission. © 2008 Elsevier.

The utilization of iFRAP to study the dynamics of mRNAs at speckles (subnuclear domains localized at the interchromatin region, containing pre-mRNA splicing factors) exemplifies both the procedure and potential of iFRAP. This work mainly addressed the question on the physiological meaning of the accumulation of transcribed mRNA in these speckles. To answer this highly relevant biological question, fluorescently labeled *Drosophila fushi tarazu* (ftz) pre-mRNAs were micro-injected into the nuclei of Cos7 cells and the association and dissociation kinetics of pre-mRNAs from these speckles were analyzed using iFRAP as shown in Figure 14. The authors showed that some pre-mRNAs can be shuttled between speckles and the nucleoplasm, suggesting that pre-mRNAs repeatedly associated with and dissociated from speckles until introns were removed (Figure 14) [127].

#### 2.1.4. Summary of the Steps to Perform in FRAP Experiments

**1)** Definition of the cell region to be bleached (ROI).
**2)** Acquisition of control images to measure intensity before bleaching.
**3)** Brief illumination of the bleach region with very high laser intensity. Ideally the bleaching event should be ultra-short, followed by subsequent image acquisition without time delay.
**4)** Recording the progress of fluorescence recovery in the bleached area with high temporal resolution.
**5)** Changes in intensity in the bleached region represent the sum of all movements of the fluorescent molecules, whether passive (*e.g.*, diffusion) or active (*e.g.*, transport).

The regeneration time (half-recovery period) is a measure for the speed of protein movement. 

#### 2.1.5. Potential Complications and Pitfalls

Apart from erroneous data modeling and processing or operator errors, there are several potential and important complications associated with FRAP [122], some of which are unexpected and should be taken into account:

1) Living cells often move during the experiment, thus after the experiment and before the regions for analysis are defined, it is recommended to use an ‘alignment’-algorithm to compensate for these movements.
2) As the total amount of excitable fluorochromes present in the cell or structure under examination is reduced over time through the bleaching event, a control region must be measured and the recovery curve must be corrected for the overall loss in fluorescence.
3) When bleaching a region in a three-dimensional sample, fluorochromes above and below the focal plane are also bleached. The bleached volume can only be assumed to have a conical shape if microscope objectives with a low numerical aperture are used. It should be accounted for that in most cases when objectives with high numerical aperture are applied, the bleached structure is far more complex than visible in the focal plane.
4) In some instances the final FRAP result is determined by the size of the ROI. It is therefore important to include a control to exclude this.
5) If low levels of fluorochromes are present, a higher intensity is needed to obtain sufficient signal. Corrections for potentially high acquisition bleaching may result in incorrect FRAP results when an immobile fraction is present. If an immobile fraction is present, correction is difficult, because the immobile fraction contributes more to the loss in fluorescence than the mobile fraction. The immobile fraction is continuously illuminated, unlike the mobile fraction which has more freedom and diffuses freely.
6) When bleached and fluorescent molecules exchange with compartments distant from the bleach region, a secondary recovery will be recorded that partly overlaps the initial recovery. This leads to an apparent slowdown of the proteins’ mobility and a general underestimation of the mobility, which is especially problematic when proteins accumulate in foci, *e.g.*, during DNA damage repair.
7) Fluorochrome intermittency (blinking) or reversible photobleaching may cause flawed FRAP results. This is especially a problem in FPs, since it has been shown that several of these, foremost GFP, rapidly switch between a dark non-fluorescent state and a fluorescent state [128], which causes an apparent erratic stroboscopic effect. The time that GFP spends in the dark state is independent of the laser settings, whereas the fluorescent state is distinctly dependent on the settings [129,130]. Partially the fluorescence recovery after bleaching is caused by the decreased number of fluorochromes in the dark state, since the bleach pulse is much higher in intensity than the monitoring after bleaching [122].
8) Because photo-induced cross-linking may occur (free radical induced cross-linking reactions), it is important to check the dependence of the recovery rate on different bleaching intensities.
9) Repeating FRAP on the same spot constitutes an important control to exclude differences in the FRAP result due to photo-damage. A higher recovery shows the presence of a “real” immobile fraction, whilst a similar recovery indicates photo-damage.

**Figure 15:**
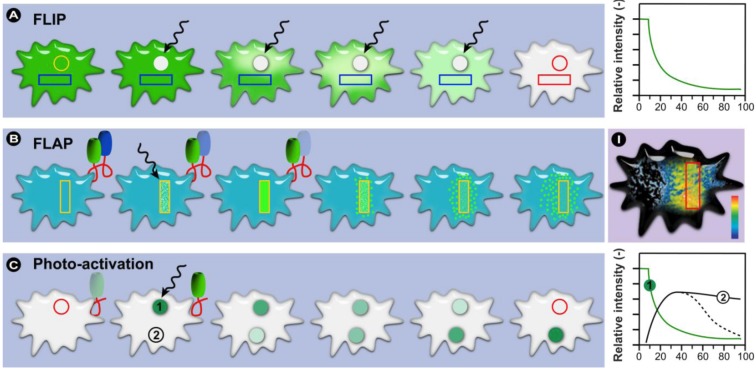
Schematic representation of FLIP, FLAP, and PA experiments. (**A**) FLIP experiments involve repetitive bleaching of a selected ROI during the entire monitoring period and the fluorescence intensity in regions outside the selected bleached area is measured. The decline in fluorescence intensity in the surrounding regions is due to bleaching of fluorochromes that move through the ROI during the repetitive bleaching process. The drop in fluorescence intensity outside the bleached region is caused by a steadily increasing population of bleached, non-fluorescent molecules within the cell and thus provides quantitative data on their molecular mobility. (**B**) In FLAP, a protein is tagged with two fluorescent labels: one is photobleached and the other acts as a reference. The use of a reference fluorochrome allows the tracking of the distribution of the labeled molecules by simple image differencing (**I**) and thus enables measurement of fast relocation dynamics. (**C**) In photo-activation (PA), the passive fluorochrome (non-fluorescent) is activated with an appropriate wavelength, which removes any quenchers or realigns bonds so that the active chromophore is formed. The loss in fluorescence in compartment 1 and gain in 2 are monitored simultaneously, which provides information on protein dynamics and compartment interconnectivity. Note that when the fluorochrome moves from 1 to 2 and subsequently diffuses out of that compartment, the curve reaches a maximum and decreases again (dotted black line).

### 2.2. Fluorescence Loss in Photobleaching (FLIP)

#### 2.2.1. The Basic Principles of FLIP

A complementary technique to FRAP, termed fluorescence loss in photobleaching (FLIP), has been used to reveal the connectivity between different compartments in the cell or the mobility of a molecule within the whole compartment [113,131]. FLIP experiments differ from FRAP and iFRAP by the repetitive bleaching of the same region in the specimen (Figure 15A), thereby preventing recovery of fluorescence in that region. 

**Figure 16:**
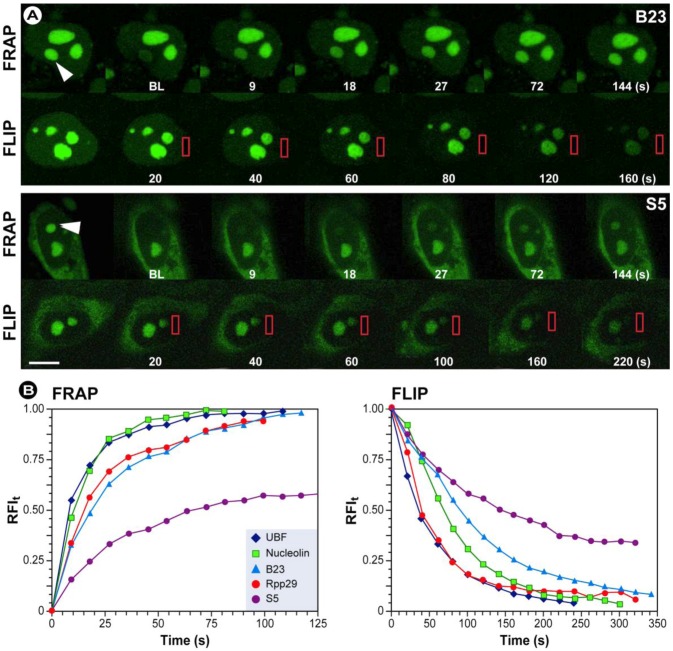
Combined FRAP and FLIP experiments assess the difference in mobility of nucleolar and ribosomal proteins in transit between the nucleolus and nucleoplasm. (**A**) In the FRAP panel the arrows indicate the sites of bleaching (ROI). In the FLIP panels the red rectangles indicate the area to be bleached. BL is the first image obtained immediately after photobleaching. Bar, 10 μm. (**B**) The FRAP and FLIP analysis plots show that the GFP-labeled ribosomal protein (S5) exits the nucleolus slower than the nucleolar factors B23, UBF, Nucleolin and Rpp29. Adapted from [132] with permission. © 2001 Rockefeller University Press.

In FLIP experiments the repetitive bleaching occurs adjacent to the unbleached ROI (Figure 15A). The loss in fluorescence in the ROI defines the mobile fraction of the fluorescently labeled protein. Conversely, the incomplete loss in fluorescence defines the immobile fraction of fluorescently-labeled protein that does not move into the continuously photo-bleached area. The observation that molecules do not become bleached suggests that they are isolated (immobilized) in distinct cellular compartments. FLIP experiments are very useful to demonstrate the connectivity and fluxes between different regions of the cell and thus is an ideal and direct method for studying the exchange of molecules between two compartments (*e.g.*, compartments that are separated by lipid bilayers). The continuity of cellular structures, such as the Golgi apparatus, the endoplasmic reticulum, the protein traffic between the nucleus and cytoplasm, the nucleolus and splicing factor compartments, and the nucleolus and nucleoplasm have all been studied using FLIP [131,133,134,135]. FLIP is often used in combination with FRAP experiments to obtain combined information regarding active or passive transport. In fact, FLIP can be used as a control for FRAP experiments. 

For example, FRAP and FLIP were used in conjunction to determine the mobility of GFP-tagged proteins involved in various steps of ribosome biogenesis in living cell [132]. The comparative FRAP and FLIP analysis of the protein dynamics data (Figure 16) revealed that the nucleolar proteins, upstream-binding factor-1 (UBF1), nucleolin, fibrillarin, Rpp29 (a human RNase P subunit), and B23 (multifunctional nucleolar phosphoprotein) move much faster between the nucleolus and nucleoplasm than the ribosomal proteins S5 and L9. The FLIP-FRAP investigations by Chen and Huang [132] suggest that a new level of regulation for rRNA synthesis exists. Furthermore, by combining FRAP and FLIP, the different dynamical properties of the proteins involved in the various steps of ribosome biogenesis could be determined and discriminated (Figure 16B). The results imply that the proteins’ nucleolar association is likely due to their specific functional activities rather than specific nucleolar-targeting events.

#### 2.2.2. Summary of the Steps to Perform in FLIP Experiments

1) Definition of the cell region to be bleached (ROI).
2) Acquisition of control images to measure intensity before bleaching.
3) Brief repeated illumination of the bleach region with very high laser intensity.
4) Recording the progress of fluorescence decay in the adjacent non-bleached area with high temporal resolution, ideally simultaneously with bleaching.
5) Changes in intensity in the non-bleached region represent the sum of all movements of the fluorescent molecules, whether passive (*e.g.*, diffusion) or active (*e.g.*, transport).
6) The decay time (half-decay period) is a measure of the speed of protein movement.

The complications and complexities that need to be considered in a FRAP experiment apply in a similar way to FLIP experiments and need to be taken into account. These were described in more detail before (*vide supra*).

### 2.3. Fluorescence Localization after Photobleaching (FLAP) and Photo-Activation Methods

FRAP and FLIP are highly efficient tools to study the dynamics of unbleached molecules and are increasingly being used to elucidate fundamental biological questions. However, the tracking of all labeled molecules is not possible by these two photobleaching methods, since the bleached molecules can consequently not be visualized. A number of alternative approaches have been developed to overcome this limitation. For example, the use of caged fluorochromes [136,137], photo-activatable or photo-convertable fluorescent proteins [138], and the development of techniques such as fluorescence loss after photo-activation (FLAC) [139] and fluorescence localization after photobleaching (FLAP) [140] have made individual tracking of fluorochromes and labeled proteins possible with high spatio-temporal resolution. A major advantage of FLAP and photo-activation/conversion techniques is that they allow the detection and tracking of sub-populations that move rapidly and have short residence times. Such studies would not be possible using FRAP or FLIP. Furthermore, the technique of photo-decaging fluorescent probes cannot be applied to fluorescent proteins, which are directly expressed in living cells and thus FLAP and related techniques offer major advantages over more conventional methods.

Fluorescence localization after photobleaching (FLAP) is a technique initially developed by Graham Dunn [140,141], in which the (bio)molecule of interest carries two fluorescent labels. One label is locally bleached, whilst the second remains intact and is used as a reference label (Figure 15B). Both fluorochromes can be imaged independently or simultaneously by fluorescence microscopy. In order to obtain reliable FLAP results, the gain and offset for each fluorescence channel should be optimized in such a way that the two images are closely matched without saturation. The absolute FLAP signal is obtained by subtracting the bleached signal from the unbleached one, allowing the tracking of the labeled molecule. A relative FLAP image can also be calculated to show the photobleached fraction of molecules within each pixel. This particular information is not available with other methods. 

**Figure 17:**
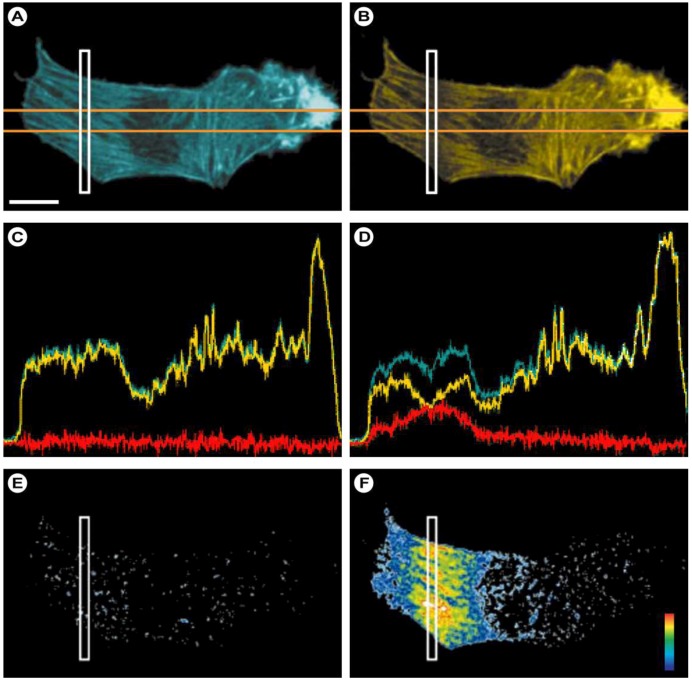
Transformed rat fibroblast showing simultaneously acquired CFP (**A**) and YFP (**B**) images immediately after photobleaching the lamella in a narrow strip (white rectangle). Intensity profiles integrated between the orange lines before (**C**) and after (**D**) photobleaching show CFP (cyan), YFP (yellow) and FLAP (red) signals. The FLAP images corresponding to the profiles (**C**) and (**D**) are shown encoded in pseudocolor in (**E**) and (**F**). Bleach time 3.8 s. Scale bar: 10 µm. Reproduced from [140] with permission. © 2002 Elsevier.

FLAP as a techniques is exemplified in Figure 17, which shows a rat fibroblast in which cDNA fusion constructs of β-actin with yellow (YFP) and cyan (CFP) fluorescent proteins were microinjected into the nucleus to study the actin filamentous turnover [140,141]. This study, apart from establishing FLAP as a technique, revealed that monomeric (globular) G-actin displayed much faster relocation dynamics than filamentous F-actin. In other investigations, Gerlich *et al.* [142] used stable expression of CFP- and YFP-tagged histone H2B molecules in normal rat kidney (NRK) cells to follow these throughout the cell cycle by 4D imaging. Their experiments showed that during interphase, in G1, S, and G2 phase of the cell cycle, no global chromosome rearrangements occurred, but observed a striking order of chromosomes throughout mitosis, which suggests that global chromosome positions are heritable through the cell cycle in mammalian cells. These experiments strikingly illustrate FLAP’s unique capacity to discriminate populations that move at different speeds and with dissimilar dynamics.

As an extension of FLAP, more recently photo-activatable [125] and photo-convertable [143,144] fluorescent proteins were developed, which allow activation or fast fluorescence switching by selective illumination with specific wavelengths. In photo-activation (PA), a fluorescent label, often a fluorescent protein, is irreversibly activated from a low fluorescent (dark) state to a bright fluorescent one by irradiating the sample with light of a specific wavelength, intensity and for a particular duration (Figure 15C). The change in fluorescence intensity is monitored in both the compartment in which the probes are activated (compartment 1) and the destination compartment (compartment 2). 

Even though these procedures are similar to iFRAP, photo-activation offers the advantage that the entire cell does not need to be bleached and consequently requires less energy and time to start the experiment. Furthermore, gross bleaching always carries with it the potential to induce oxidative stress, which constitutes a significant and additional deviation from normal cellular homeostasis. In addition to the aforementioned advantage, fast moving sub-populations can be detected, unlike in iFRAP where these fluorochromes are bleached and thus remain undetectable. Examples of photo-activatable proteins are photo-activatable GFP (PA-GFP) [125] and Dronpa [145], which have been expressed in living cells to study numerous cellular processes. Fluorescent proteins such Dronpa, PA-GFP, rsCherryRev, and IrisFP, are fluorescent to begin with, but undergo quenching when illuminated with a specific wavelength, whilst fluorescent proteins such as KFP, rsCherry, and rsTagRFP, are initially non-fluorescent and activated to a short-lived fluorescent state upon illumination with an appropriate wavelength [115].

Photo-conversion or switching follows slightly different principles. Photo-convertible proteins like Kaede are irreversibly converted from green to red fluorescent with a pulse of ultraviolet light [143]. Thus, unlike photo-activation, the fluorochrome changes its fluorescence color. This allows comparable measurements as with FLAP, but similar to photo-activation requires less energy and less time to initiate the experiment. Nonetheless, analogous to photo-activation, photo-conversion is irreversible, which limits repetitive measurements to determine alterations in protein mobility in response to cellular challenges or other external events or signals, since in photo-conversion the number of repetitive experiments is limited by the amount of photo-convertible material that is available. Additional major drawbacks of virtually all photo-activatable and convertible proteins are the fact that they generally require ultraviolet light for activation and that proteins such as PA-GFP and photo-activatable monomeric red fluorescent protein-1 (PAmRFP1) require higher laser intensities for photo-activation. *In toto*, such experimental settings might still induce significant phototoxicity, especially during long-term acquisition and experimentation, and are generally unsuitable for processes in which changes in the redox-state are essential. To overcome such limitations, photochromic fluorochromes have been introduced, which allow selective and reversible switching between a fluorescent and dark state induced by light of the appropriate wavelength, one of these is kindling fluorescent protein-1 (KFP1) that can be activated with green wavelengths and thus shows significantly lower phototoxicity. Alternatively, multi-photon activation basically circumvents the necessity to use an activation pulse in the UV. Besides progression in the generation of photoswitchable proteins with novel properties, recent advances in nanoparticle synthesis produced photoswitchable nanoparticles with either fluorescence on/off or dual-alternating-color fluorescence photoswitching for use in a myriad of cell biological applications [146]. One limitation remains in photoswitchable fluorescent proteins: FPs such as asFP595 or Dronpa ultimately fade after a number of switching cycles and cannot be activated again [147,148]. Stefan Hell’s group now report a photochromic variant of EGFP, reversibly switchable enhanced green fluorescent protein (rsEGFP), which could be reversibly switched “ON” at λ = 405 nm and “OFF” at 491 nm and can undergo more than a thousand switching cycles [149]. Such advances will benefit FRET-, localization-, diffusion-, and super resolution-based studies. Fluorescent proteins that are activatable or switchable are required for super-resolution imaging of live cells and can be well-controlled in a spatio-temporal manner. One remaining major limitation of photoswitchable proteins is the fact that the wavelengths for switching and fluorescence imaging are generally coupled. Again, Hell’s group resolved this problem by introducing a bright photochromic variant of GFP, Dreiklang, whose fluorescence excitation spectrum is decoupled from optical switching and allows reversible switching at illumination wavelengths of ~365 nm and ~405 nm, respectively, whereas fluorescence is elicited at ~515 nm [150]. Dreiklang can effectively be used for nanoscopy and fluorescence recovery after switching.

## 3. Energy Transfer Methods for Inter- and Intra-Molecular Interaction Measurements

### 3.1. Förster Resonance Energy Transfer (FRET)

**Figure 18:**
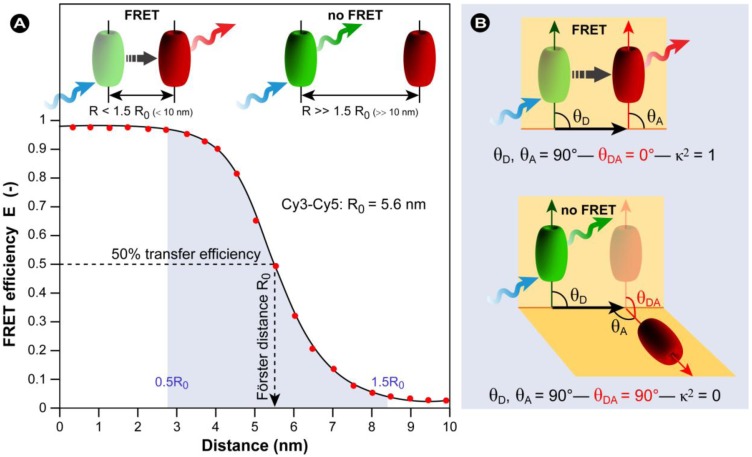
Schematic representation of FRET as a photophysical process. FRET depends on the close proximity of a donor and acceptor pair of fluorescent molecules in which the emission spectrum of the donor overlaps the excitation spectrum of the acceptor. Because these molecules must be in proximity of less than 10 nm for FRET to occur, the spatial resolution of the microscope is significantly improved. If only the donor fluorochrome is excited and the acceptor molecule is too distant from it, only donor fluorescence will be detected. However, when the acceptor is closer to the excited donor, energy can be transferred non-radiatively from donor to acceptor (FRET). The intensity of donor emission decreases, while an acceptor emission can be distinctly detected.

FRET is a process in which energy is transferred non-radiatively (that is, via long-range dipole-dipole coupling) from an excited donor fluorochrome to another molecule or acceptor. The acceptor does not necessarily need to be fluorescent. FRET relies on the close physical interaction of the two molecules (donor and acceptor) and can only occur if the distance between donor and acceptor is less than approximately 10 nm [151]. Thus, FRET can be used to determine molecular interaction/molecular proximity beyond the resolution limits of the classical light microscope (Figure 18).

The theory of ‘resonance energy transfer’ was first postulated by Theodor Förster [15,16] and in honor of his contribution, the effect was named after him. However, the term fluorescence resonance energy transfer is now commonly used in the scientific literature. This is strictly taken misleading, because the process always involves non-radiative transfer of energy, even between two fluorescent chromophores. 

In the original work, Förster derived an expression for the rate constant of transfer *k_T_* as follows: 


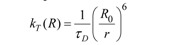
 (14)

**Figure 19:**
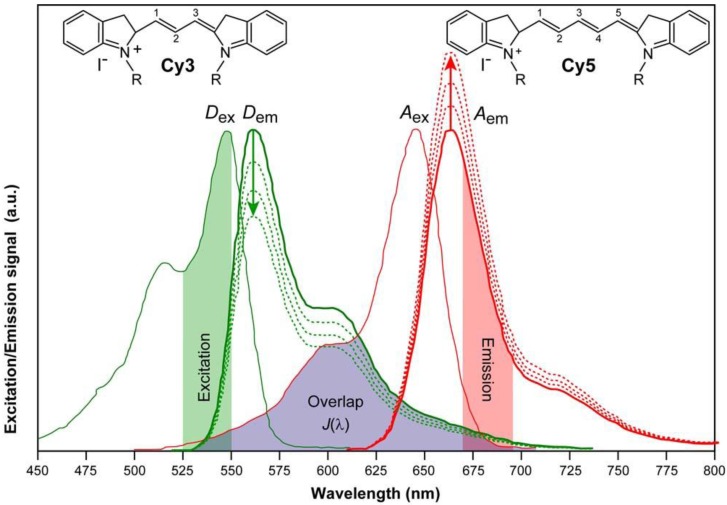
Overlap integral of a FRET pair. In this case the overlap of the Cy3 emission spectrum and the excitation spectrum of Cy5 are depicted as an example. As a result of FRET, the donor emission (*D*_em_) is reduced while the acceptor emission (*A*_em_) increases.

The Förster theory of energy transfer states that the efficiency of the energy transfer, commonly denoted the FRET efficiency (*E_FRET_*), depends on the physical distance between donor and acceptor, the spectral overlap of the donor emission spectrum and the acceptor absorption spectrum (Figure 19), and the relative orientation of the donor emission dipole moment and the acceptor absorption dipole moment (Figure 18B). The efficiency *E_FRET_* depends on the donor-to-acceptor separation distance (*r*)and scales with an inverse 6^th^ power law due to dipole-dipole coupling according to:


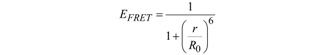
 (15a)

or:


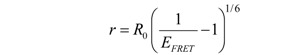
 (15b)

The Förster radius (*R*_0_) is the characteristic distance where 50% FRET efficiency occurs, which can be calculated from the spectroscopic and mutual dipole orientational parameters of the donor and acceptor. *R*_0_ defines the length scale of the interaction. The effective yield range of *R*_0_ is about 3–8 nm, which corresponds to the 5%–95% range of *E_FRET_* where changes can still be detected with sufficient sensitivity (0.5*R*_0_–1.5*R*_0_ in Figure 18A). In practice the useful range is smaller (*e.g.*, 4–7 nm) due to experimental limitations such as noise. With organic fluorochromes or with fluorescent proteins the value of *R*_0_ is generally around 5 nm and for the FRET couple Cy3/Cy5 in Figure 18A, *R*_0_ = 5.6 nm and consequently a sufficiently high FRET efficiency can be achieved at inter-chromophoric distances below 8.4 nm. At such close distances, the occurrence of FRET provides proof that the donor and acceptor associate closely. However, the relatively short working distance of FRET is a limitation for studying multiprotein complexes or interactions between very large proteins. 

As stated previously, the Förster distance depends on the overlap integral of the donor emission spectrum with the acceptor absorption spectrum and their mutual molecular orientation (Figure 18B and Figure 19). The *R*_0 _value in an aqueous solution is determined by an equation with known input parameters:


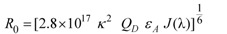
 (nm) (16)

where *Q_D_* is the fluorescence quantum yield of the donor in the absence of the acceptor, *ε_A_* is the maximal acceptor extinction coefficient (mol^-1^ cm^-1^), and *J*(λ) is the spectral overlap integral between the normalized donor fluorescence, *f*_D_(λ), and the acceptor excitation spectra, *ε_A_*(λ): 


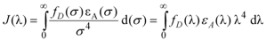
 (17)

The dipole orientation factor *κ^2^* is given by:


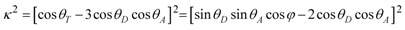
 (18)

This equation describes, as depicted schematically in Figure 18B, that FRET coupling directly depends on the angle between the two fluorochromes. As shown in Figure 18B, if the donor and acceptor are aligned parallel to each other, the orientation is ideal and the FRET efficiency will reach near maximal values, whereas in a perpendicular orientation, the FRET efficiency is virtually negligible. This degree of alignment defines the size of *κ^2^*. While *κ^2^* can vary between 0 and 4, it is usually assumed to be ⅔, which is the average value integrated over all possible angles. These values are obtained when both fluorochromes have the maximal degrees of freedom, *i.e.*, are freely rotating and can be considered to be isotropically oriented during the excited state lifetime.

If one of the fluorochromes is immobilized or does not freely rotate, then *κ*^2^ = ⅔ will not be a valid assumption. In most cases, however, even modest reorientation of the fluorochromes results in sufficient orientational averaging and assuming *κ*^2^ = ⅔ will not induce a significant error in the estimated energy transfer distance due to the sixth power dependence of *R*_0_ on *κ*^2^. Even when *κ*^2^ is quite different from ⅔, the error is often associated with a shift in *R*_0_ and thus determinations of changes in relative distances for a particular system are still valid. Fluorescent proteins do not reorient on a timescale that is faster than their fluorescence lifetime—in this case 0 ≤ *κ*^2^ ≤ 4. Experimentally, Stryer and Haugland confirmed Förster’s distance postulate with an α-naphthyl donor group at the carboxyl and a dansyl acceptor group at the imino end of variable length poly-L-proline oligomers [152] and later also confirmed the overlap requirement [153].

Finally, it needs to be pointed out that because of the spectral overlap, which is a prerequisite for FRET to occur, the FRET signal is always affected by donor emission into the acceptor channel and by the excitation of acceptor molecules by the donor excitation wavelength (spectral bleed-through [SBT]). Furthermore, FRET signals in the acceptor channel must also be corrected for external and instrument signals, such as background fluorescence from intrinsic (bio)fluorochromes, instrument noise (optics, detector, etc) and spectral sensitivity variations in donor and acceptor channels, which contaminate the FRET results. Algorithms and spectral unmixing are generally used to remove unwanted signals from the FRET signal and the process of removing SBT is described extensively in the literature (see references [154,155,156,157,158]). An ImageJ plug-in for FRET calculation that can accommodate variations in spectral bleed-through was recently developed by Feige *et al*. [159] and other routines are available from the NIH-RSB website (http://rsbweb.nih.gov/) for both ImageJ and NIH Image.

**Figure 20:**
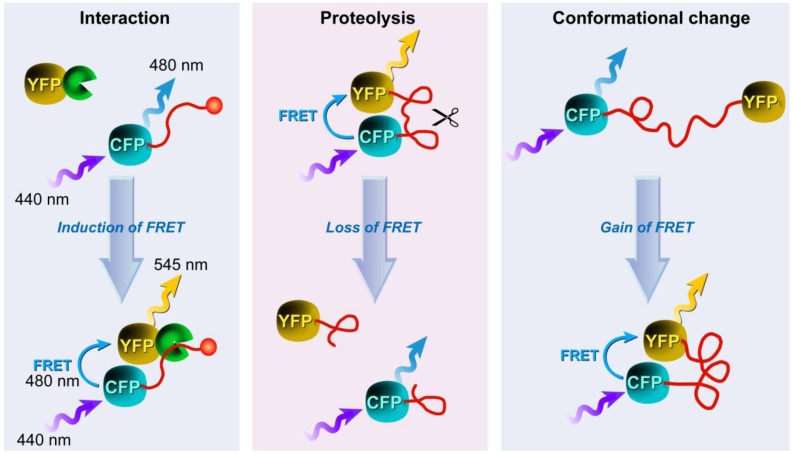
Three possible approaches for developing FRET biosensors. Based on [183].

Fluorescent proteins and organic fluorescent dyes have been successfully used as FRET pairs in the past, either as single labels of different proteins or intra-molecularly to study structural changes (Figure 20). The wide range of genetically modified forms of GFP and the identification of Anthozoan and non-Cnidarian animal-derived variants resulted in a broad palette of FPs virtually spanning the visible spectrum. There are a number of different FRET pairs that can be used depending on the biological application and scientific question to be resolved. A major criterion in the selection of a suitable FRET pair is the Förster distance *R*_0_. The probability that FRET occurs increases with increasing spectral overlap *J*(λ), Förster distance *R*_0_, donor quantum yield *Q_D_*, and the acceptor’s extinction coefficient *ε_A_* (these properties are listed for a number of pairs in Table 2).

### 3.2. FRET Couples

Even though organic fluorochromes lack the benefit of genetic coding and direct expression in the cell, they nonetheless offer several unique advantages over FPs. In particular the red emitting dyes (>500 nm), such as the cyanine dyes Cy3, Cy5, Cy5.5 and Cy7, various BODIPY and Alexa dyes, have emission ranges outside the autofluorescence window, offer higher photon counts per fluorochrome molecule compared with the relatively dim FPs [160,161], and have higher extinction coefficients. ATTO dyes for instance show excellent photostability and brightness and ATTO 647N fluoresces nearly twice as strong as Cy5 in aqueous solution. Consequently, large donor-acceptor distances above 10 nm can still be measured and even if the FRET pairs have a low overlap integral, acceptable results can be obtained – the Cy3/Cy5 couple in Figure 19 has a Förster distance of 5.6 nm. Furthermore, such large FRET pair separations allow the measurement of the acceptor without much interference by the donor. For these reasons, organic dyes retain their value and popularity in biological research. Equally, lanthanide-based chelates (*e.g.*, Eu(III), Tb(III) and Sm(III)) enjoy some popularity, because they have long fluorescence lifetimes in the (sub)millisecond range [5,7] and as such are ideal donors for time-resolved FRET measurements. Because their lifetimes are much longer than the lifetime of organic dyes and biological fluorochromes, autofluorescence and other unwanted signals can easily be eliminated and consequently time-resolved imaging drastically increases FRET sensitivity [7,162,163].

**Table 2:** Overview popular organic dye and fluorescent proteins FRET couples and some relevant photophysical properties.

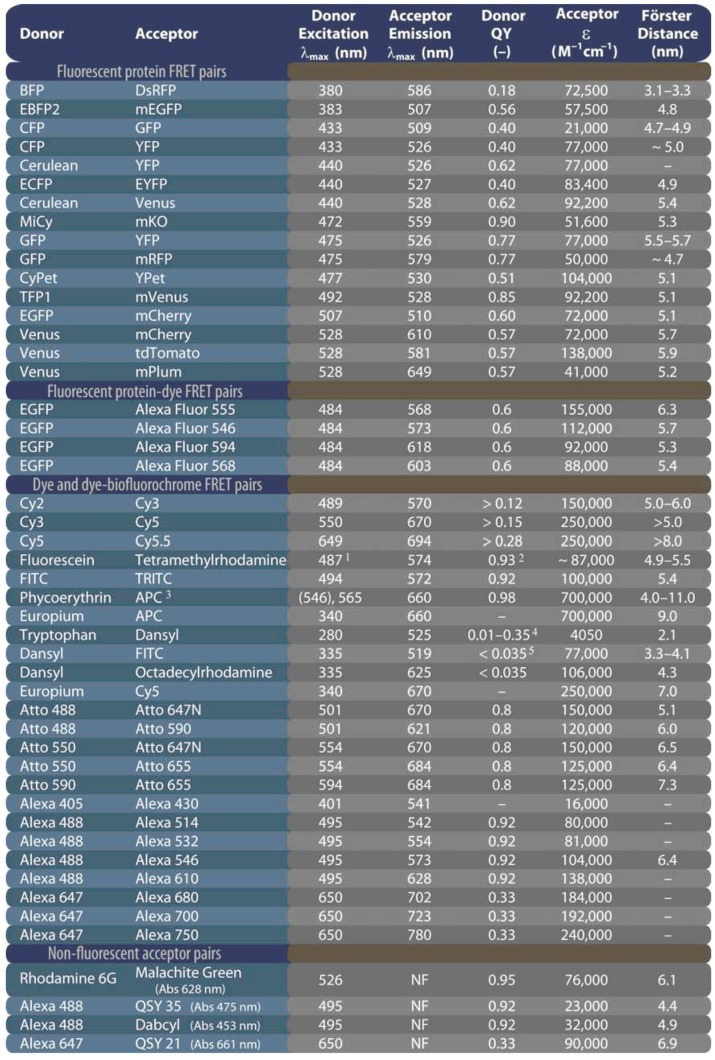

NF: Non-fluorescent; ^1^ pH > 7; ^2^ in 100 mM sodium borate buffer, pH 9.5; ^3^ Allophycocyanin is an accessory photosynthetic pigment from blue-green algae (6 phycocyanobilin chromophores/molecule). APC and Alexa 647 cannot be used simultaneously due to nearly identical excitation and emission properties; ^4^ from [164]; ^5^ from [165].

The first truly effective fluorescent protein FRET pair, devoid of problems such as poor photophysical properties and ineffective overlap integrals, consisted of CFP as the donor and YFP as the acceptor [166]. This FRET couple or their enhanced versions remain popular and widely used to date. However, it should be noted that cross-talk in CFP/YFP FRET is a major problem when using a 458 nm laser in the CLSM. At this wavelength, CFP is not optimally excited, but concomitantly the use of the 458 nm laser causes considerable direct excitation of YFP and thus SBT. 

A number of commonly used FRET couples are listed in Table 2, although these represent but a fraction of the pairs reported in the literature. Other popular FRET pairs include CFP/*Discosoma* Red (dsRED), blue fluorescent protein (BFP)/GFP, GFP or YFP and dsRED, and even combinations such as Alexa488 as donor and Cy3 as acceptor, FITC and Rhodamine, and YFP as donor and Tetramethyl Rhodamine Iso-Thiocyanate TRITC or Cy3 as acceptor. More recently, GFP or YFP as the donor coupled with orange or red derivatives, such as monomeric Kusabira Orange (mKO) [167] or mCherry [168], and an orange donor coupled with a red acceptor were used as FRET bio-sensors. The orange and red coral-derived proteins have broad excitation spectra, causing direct acceptor excitation as their key limitation. Other FPs with superior excitation coefficients and quantum yields are the optimized CFPs mCerulean [169] and SCFP3A [170], and the optimized YFPs mCitrine [171], SYFP2 [170] and mVenus [172].

A note of caution needs to be expressed with regard to donor–acceptor couples. Generally FRET theory is based on the assumption that in a FRET couple only a single donor and a single acceptor are present with very weak coupling [8,39]. In molecular complexes in living cells it is generally unknown if a single or multiple acceptors are present. In case of multiple acceptors, until now a simple kinetic model is used that assumes that the donor interacts separately with each acceptor and as such the collective FRET efficiency can be calculated from the sum of all FRET transfer rates divided by the sum of all radiative and non-radiative transfer rates [8,173]. However, a recent study by Vogel and co-workers showed an anomalous surplus energy transfer in Cerulean–Venus constructs with multiple Venus acceptors [174]. The authors speculate that “either an additional energy transfer pathway exists when multiple acceptors are present, or that a theoretical assumption on which the kinetic model prediction is based is incorrect” [174]. Even though this study does not provide any conclusive explanation for the observed phenomenon, it does show the necessity to interpret quantitative FRET experiments with care, to cautiously evaluate labeling of biomolecules with fluorochromes, and to meticulously perform control experiments.

Over the past decade, as a result of the explosive developments in the (bio)nanotechnological field, luminescent nanoparticles have been developed for use in numerous technical, chemical, physical and biological research fields and applications including FRET imaging, most notably inorganic semi-conducting quantum dots (Figure 6B). QDs offer a number of advantages (see also §1.2), but equally suffer from several disadvantages over conventional fluorochromes. As stated previously, organic dyes and fluorescent proteins largely preclude multiplexed FRET methods to measure multiple processes simultaneously because of poor spectral separation or such measurements can only be achieved with complex instrumentation and laborious spectral unmixing routines. For example, a fully validated and modeled three-color spectral FRET (3sFRET) method based on the detection of the sensitized emissions from acceptors through steady-state confocal spectral imaging microscopy was developed by Periasamy’s group. The method was used to image the interactions of the dimeric transcription factor C/EBPα (expressing mTFP or mVenus) with the heterochromatin protein-1α (expressing tdTomato) in live-mouse pituitary cells [175]. However, 3sFRET also demonstrates the difficulties involved in multiplexing FRET with the current generation of fluorochromes and FPs. Besides such unmixing complexities, a number of conventional, but suitable FRET fluorochromes suffer from poor intensities, environmental susceptibilities (pH, solvent polarity, *etc.*), and a propensity to chemical and photo-induced degradation. Conversely, QDs have narrow symmetric photoemission, which can be directly controlled by their physical size, have high extinction coefficients, show exceptional brightness, and have broad absorption spectra that steadily increase towards the UV (Figure 6C), and are photo-stabile. Their brightness and high photon output results from their particularly large extinction coefficients, which are often larger than 10^6^ M^−1^ cm^−1^ (*e.g.*, 15,800,000 for Qdot 800 at 350 nm [176]) compared with organic dyes and FPs (<10^5^ M^−1^ cm^−1^; see Table 2). The ability to simultaneously excite QD populations with multiple emission maxima with a single wavelength far removed (>100 nm) from their respective emissions allows extensive multiplexing with far less complications compared with organic dyes. However, this also causes major problems in engineering QDs as acceptors in QD-FRET, since their broad absorption spectra make it difficult to avoid direct proximity-independent excitation (SBT). Furthermore, their relatively long fluorescence lifetimes pose an additional impediment for successful FRET acceptor implementation. Clapp *et al*. perseveringly attempted to create suitable organic dye–QD FRET couples, but reported major problems and failed to observe energy transfer [177]. On the contrary, Rao and co-workers reported a bioluminescence resonance energy transfer (BRET) based method, in which COOH-modified 655 nm emitting QDs were used both as acceptor and as a scaffold to which an average of 6 molecules of an optimized variant of luciferase as donor were coupled [178]. In this way, not only energy transfer to QDs as acceptor was achieved, but since BRET does not require an excitation beam, self-illuminating far red to near-IR emitting QDs for deep tissue imaging were produced. Nonetheless, at the present time, it is unclear whether QDs can be engineered to act effectively as acceptors for organic dyes and FPs. Other disadvantaged include: (i) the proximity requirement for FRET precludes the use of large and thus red/infrared emitting QDs, since the Förster distance may fall within the core-shell radius [179], and (ii) fluorescent proteins as FRET partners offer the major advantage that they are genetically encoded and can be manipulated on the genetic level and thus QDs cannot be used for all purposes. Despite all these restrictions, numerous QD-based FRET applications in which QDs are used as donors have been reported in the literature, the majority of which are *in vitro* assays. Clapp *et al*. [180], Medintz and Mattoussi [36], and Barosso [181] provide balanced reviews on new and recent advances regarding QD-FRET and its limitations and the problems that remain to be resolved.

### 3.3. Applications of FRET in Cell Biology

FRET is a particularly powerful technique in combination with *in cellulo* expression of chimeric proteins with FPs as labels. FRET-based techniques have been used to study a myriad of biological processes, including protein–protein interactions in various cell biological settings, such as signal transduction, or conformational changes within proteins, *e.g.*, the activation of enzymes, Ca^2+^ signaling, nucleic acid studies, characterization of gene expression, and real-time PCR assays. Some of these applications utilized microscopic imaging to visualize such processes, whilst others are purely performed *in vitro* and analytical in nature. GFP-based FRET imaging methods have been a crucial tool in determining the compartmentalization and functional organization of living cells and for tracing the movement of proteins inside cells [182]. A potent application of the FRET principle is the use of FRET-biosensors, which are frequently fusion proteins of ECFP and EYFP (or other appropriate FRET pairs such as EGFP and mRFP) linked by a sensory domain. This domain responds to changes in certain cellular parameters by a conformational change (Figure 20), leading to a change of the FRET signal. The basic principle relies on the ratiometric monitoring of donor and acceptor channels and the detection of changes in the FRET signal as a result of biological activity. 

Fundamentally there are three approaches in the design of FRET-based biosensors (Figure 20): (i) interaction of the labeled proteins results in a FRET signal, which was previously not present because the separation between donor and acceptor was too large; (ii) proteolysis of an intramolecularly labeled biomolecule leads to separation of donor and acceptor beyond 10 nm and a concomitant loss in FRET signal, and (iii) an intramolecularly labeled biomolecule undergoes a conformational change upon stimulation with a ligand or binding of a substrate, resulting in an increase in FRET. Prominent examples of FRET-based substrate binding biosensors are the Cameleons, a family of Ca^2+^ sensors based on calmodulin [166]. 

Liu *et al. *developed intramolecular biosensors consisting of CFP/DsRed or GFP/DsRed, flanked by the GTPase-binding domain of p21-activated kinase1 (PAK1), or Neural Wiskott-Aldrich syndrome protein (NWASP), and full-length Rac1 or Cdc42 [184]. Such FRET-biosensors simplify procedures to identify regulatory proteins for Rho GTPases over conventional methods with high temporal-spatial resolution. Other intermolecular FRET biosensors consisted of a CFP/YFP FRET pair to detect direct intermolecular integrin interactions [185]. The results show amongst others that integrins induce local Rac-effector coupling by directing Rac to membranes and dissociating it from Rho-GDI (guanine nucleotide dissociation inhibitors). FRET biosensors have also been used to study the interactions between receptor-ligand pairs, dimerization of individual receptors, as well as transbilayer distribution of fluorescent lipid analogs and protein-mediated lipid transfer between vesicles. FRET is also used to study the structure, conformation, hybridization, and automated sequencing of nucleic acids. More elaborate reviews on FRET biosensors, their design, and applications were recently provided by Wang [186] and Frommer [187] and their co-workers, whilst Aoki *et al.* [188] focus on FRET biosensors for studying oncogene signal transduction pathways and Varghese *et al*. [189] highlight more analytical FRET applications for lab-on-a-chip devices.

What all of these methods have in common is that they only allow the study of a limited number of events in the same cell simultaneously, since imaging multiple FRET pairs remains challenging for reasons outlined before. Nevertheless, more complex biosensors based on an alternative approach called “computational multiplexing”, which refers to the integration of data from multiple independent data sets, are currently employed and being developed further. Such studies–recently reviewed by Welch *et al.* [190]–aim to obtain a complete analysis of pathway states, which would not be possible in the same cell with the current technology. However, such an approach requires both conserved experimental conditions, since the expression or microinjection of the FP biosensor can profoundly affect the pathway, and the use of spatial and temporal fiduciaries to determine the spatiotemporal relationships between activities monitored in independent experiments [190]. These requirements illustrate that such studies are challenging and require a thorough validation of the effect of each biosensor used on cellular homeostasis and morphology, but represent nonetheless novel and exciting technology, which after maturation will certainly contribute to our understanding of pathway dynamics in an unprecedented way. 

### 3.4. Approaches to FRET Imaging

Live-cell FRET microscopy can conveniently be combined with a range of techniques including fluorescence correlation spectroscopy to investigate diffusion [191], anisotropy measurements to investigate structural relationships of molecules [192,193], TIRFM for analysis of processes on cellular surfaces [194], and FLIM and super-resolution microscopy (see below). The list of techniques that have been developed to determine FRET is quite extensive. In general, all existing strategies for measuring FRET can be applied to fluorescent protein experiments, but on the basis of practical considerations, four general approaches have proven particularly useful: (i) acceptor photobleaching; (ii) sensitized emission including the use of spectral imaging; (iii) fluorescence lifetime imaging microscopy (FLIM); and (iv) fluorescence polarization imaging. 

#### 3.4.1. Donor and Acceptor Photobleaching

##### 3.4.1.1. Basic Principles

FRET can be established by measuring the bleaching rate of the donor in the presence and absence of the acceptor. This method called donor photobleaching FRET, was initially established by Thomas Jovin’s group [145,160] and is based on the notion that a fluorochrome is only sensitive to photobleaching when it resides in the excited state (Figure 4). Fluorochromes that have longer lifetimes statistically have a higher probability to suffer photo-induced damage and therefore display higher bleaching rates. Since energy transfer directly reduces the donor’s fluorescence lifetime (by depopulation of the excited state) and the photobleaching time varies inversely with the fluorescence lifetime, the reduced photobleaching rate in the presence of the acceptor can be used to calculate FRET relative to the rate in the absence of the acceptor. Because FRET-related photobleaching experiments require long timeframes, potentially affect cellular homeostasis through the formation of reactive species, and is generally not suitable for acquiring fast dynamic processes; these studies are ideally performed in fixed samples based on pixel-by-pixel analysis. Besides the fact that donor photobleaching FRET is not suitable for live cell imaging, fitting photobleaching curves involving multiple components can be challenging. Even though donor-photobleaching FRET is generally less complicated than measuring sensitized emission FRET, acceptor photobleaching offers the advantage that the same specimen serves as its own control.

**Figure 21:**
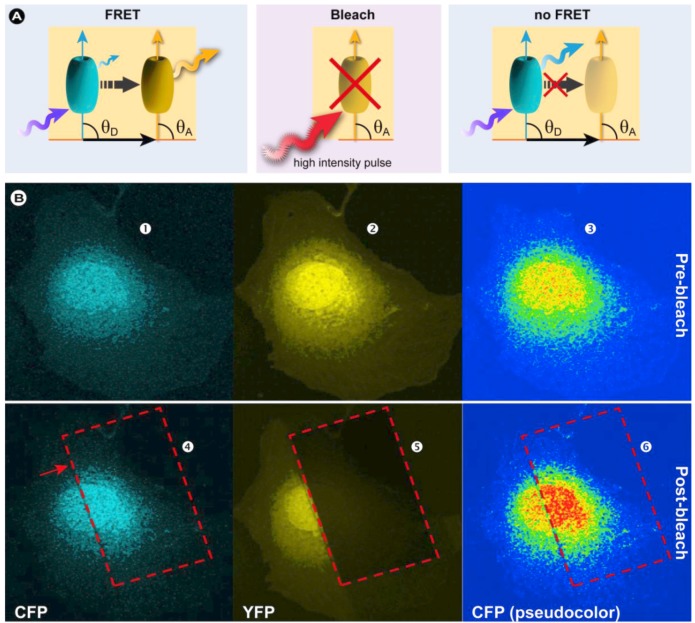
(**A**) Schematic representation of acceptor photobleaching. FRET occurs because the donor transmits part of its energy to the acceptor. As a control, the acceptor is bleached with high intensity light, until the majority of the acceptor molecules are irreversibly damaged. As a result, the donor emission will increase, since energy transfer is abolished. Depending on the efficiency of the acceptor bleaching, there will be little to no acceptor emission detectable. However, if no FRET occurred (*i.e.* donor and acceptor molecules are too distant from each other) acceptor bleaching should have no effect on the fluorescence intensity of the donor molecule. (**B**) Example of acceptor photobleaching FRET, obtained via CLSM in a living HeLa cell co-expressing an eCFP (donor) and eYFP (acceptor) construct. CFP and YFP images were excited sequentially at 458 and 514 nm and emission recorded with adequate filter sets (①–③). A region of the cell (red rectangle) was photobleached at 514 nm for 5 s ⑤. Post-bleach images were captured simultaneously at 458 nm excitation (④–⑥). FRET is visualized as an increase in CFP fluorescence ④ following YFP photobleaching ⑤, which is more prominently visualized in the pseudo-colored image (compare③ and ⑥, which shows a distinct increase in red intensity values in the nucleus). Image courtesy of B. Giese and G. Müller-Newen, Institute for Biochemistry, RWTH-Aachen, Germany.

In a typical acceptor photobleaching FRET experiment, the fluorescence intensities of the donor fluorochrome are measured before (*I*_DA_) and after photobleaching of the acceptor (*I*_D_) in a limited area (Figure 21). The difference between these donor intensity measurements enables the calculation of the FRET efficiency [195] according to:


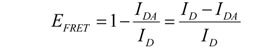
 (19)

In case energy transfer takes place, the donor fluorescence increases in the bleached area (Figure 21B). In principle, also a donor photobleaching would be possible. In the case that FRET occurs, the acceptor’s fluorescence should also vanish. In practice, donor photobleaching is not very popular as often the structure of interest is not visible any longer. 

The advantage of the acceptor bleaching approach is that it is relatively straightforward and can be carried out on any fluorescence microscope with appropriate filter sets and a powerful enough light source to bleach the acceptor. Furthermore, acceptor photobleaching requires only a single specimen preparation. However, the disadvantage of this method is that photobleaching the acceptor can also cause photo-damage to the sample. In live cell studies, there is a significant probability that the FRET measurement will be invalidated by recovery of the acceptor fluorochrome (like in a FRAP experiment) in a relatively short time. In practice, for many studies, this precludes the use of this method and means that acceptor photobleaching FRET is especially inappropriate in live cell experiments where freely diffusible molecules are under investigation [196]. Furthermore, photoconversion artifacts have been reported for acceptor photobleaching FRET experiments with CFP and YFP [197] and more recently Kremers *et al*. observed blue-shifted photoconverted species for mVenus and minor spectral shifts in mPlum and mRaspberry [198]. Such reports underscore that the complex photophysical mechanisms involved in fluorescence microscopy and the fluorochromes used, especially fluorescent proteins, require a healthy need for wariness and control experiments to prevent serious misinterpretation of results. Recently, Gadella Jr. *et al.* pointed out that the occurrence of obscured artifacts from imaging just donor intensities before and after acceptor photobleaching can be circumvented by gradual acceptor photobleaching [199]. 

The authors indicate that by measuring the donor intensity as a function of time during acceptor photobleaching, both inadvertent donor photobleaching and the presence of any background intensity can be detected without the need for additional measurements. For a detailed description of the method and algorithms used to correct and fit data see reference [199].

**Figure 22:**
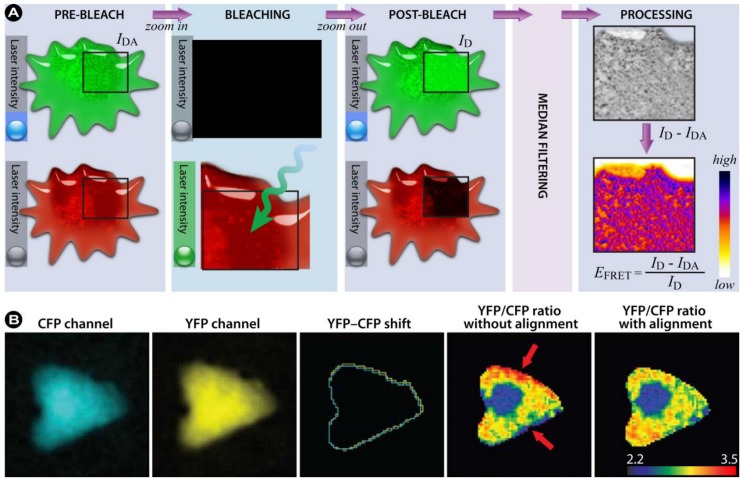
(**A**) Workflow of acceptor photobleaching FRET. (**B**) Ratio imaging artifacts. Shown is an artifact resulting from the ratio calculation of misaligned CFP and YFP images. The CFP and YFP images of a cell expressing the RacFRET biosensor are shown. For clarity reasons, the contours of the CFP and YFP images are shown to demonstrate the shift between the two channels. Arrows indicate the areas at the edges where the YFP/CFP ratio signal is incorrect as a result of the shift between the YFP and CFP images. Part B reprinted from [200] with permission from Macmillan Publishers Ltd: Nature Protocols, © 2011.

##### 3.4.1.2. Summary of the Steps to Perform in Acceptor-photobleaching Experiments

A schematic workflow of the steps in acceptor photobleaching FRET is shown in Figure 22A.

1) Choose an appropriate FRET couple to perform the experiments.
2) Acquire images of the donor in the presence of the acceptor (*I*_DA_) and of the acceptor at low laser intensity (pre-bleach).
3) Draw a ROI within the image, corresponding to the bleaching area and the part in which the FRET efficiency will be calculated
4) Zoom in on the ROI and photobleach the acceptor with high laser intensity.
5) Zoom out to the original magnification and re-record the donor (*I*_D_) and acceptor images (post-bleach).
6) By utilizing an algorithm that corrects for SBT and other unwanted artifacts, the FRET signal can be consolidated. Furthermore, background subtraction, filtering, and noise reduction will improve image quality.

*Intermezzo*: A major artifact in ratio imaging and FRET efficiency calculations, not readily noticed by the inexperienced microscopist, consists of incorrect alignment of the individual channel images. Even sub-pixel misalignment between the images during the calculation of the FRET efficiency can lead to significant errors, most often in the region between the medium and the cell border. However, edge artifacts can be virtually eliminated or reduced to a minimum by using a cross-correlation routine to align pre- and post-bleach images before FRET efficiency is calculated. Appropriate plugins are available for ImageJ and NIH Image from the NIH-RSB website (http://rsbweb.nih.gov/). The significance of misalignment when calculating ratios is demonstrated in Figure 22B, which clearly shows that minor shifts can induce dramatic errors (see red arrows).

7) Use cross-correlation to align the images.
8) Calculate the FRET efficiency according to Equation 19

In order to validate and specify the interactions measured by FRET, control experiments that characterize co-localized non-interacting proteins or mutant proteins in which the interaction is deliberately prevented by inducing the right mutation need to be performed (preferably under the same conditions and with equal expression levels).

#### 3.4.2. Sensitized Emission

An alternate way to measure FRET is by determining the sensitized emission. In this method only the donor molecule is excited, which transfers energy to the acceptor causing it to enter an excited state instead, and changes in the fluorescence signal are subsequently measured in the acceptor channel only. This is the simplest way to measure FRET and would be the ideal method if the donor and acceptor channels would be fully separated and no cross-talk would occur. Unfortunately, in practice this approach necessitates particular precautions with respect to cross-talk or “bleed-through” of the detection channels and consequently sensitized-emission FRET requires high quality, suitable and specific filters and the appropriate set of controls. In order to obtain accurate FRET results, three samples have to be prepared: (i) a sample that consists of the donor-only; (ii) the second sample consists of the acceptor-only; and (iii) the third is the actual FRET experiment sample. These samples are imaged with the respective image channel settings for the donor, acceptor, and for the final sensitized emission measurement, *i.e.*, donor excitation and acceptor detection. From these three images, the final FRET signal can readily be calculated, but adequate quantization remains nonetheless challenging [201].

There are different methods to calculate the final FRET signal (*F* *^c^*). All these methods have in common that correction factors have to be determined. 

In the Youvan method [202] the corrected FRET signal (*F* *^c^*) is calculated according to:


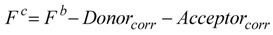
 (20)

*F* *^c^* is calculated by subtracting the corrected donor contribution (*Donor_corr_*) and the corrected acceptor (*Acceptor_corr_*) contribution from the measured FRET signal. The correction values are determined in detail by:


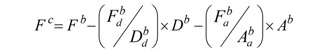
 (21)

where *D*, *A*, and *F* represent a monochrome image acquired through the donor, acceptor or FRET channel. Superscript *^b^* indicates that the monochrome image has been background subtracted and the individual pixels from the donor and acceptor fluorochrome are denoted with subscript *_d_* or *_a_*. Therefore, 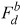 represents a background-subtracted donor image taken in the FRET channel; 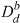 is a background-subtracted donor image taken using the donor channel. The same notation is used for the acceptor fluorochrome and the ratios in Equation 21 are used to correct the FRET channel intensities pixel-by-pixel. In this method the *F* *^c ^* is corrected for the donor and acceptor contribution to the final signal measurement with the FRET settings, but is not normalized for donor and acceptor concentrations. The method proposed by Gordon *et al.* [203] extends the Youvan method and corrects for the concentration of the donor and acceptor. The Gordon FRET correction can therefore be expressed as:


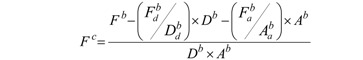
 (22)

The Gordon method works best in cases where low concentrations of donor and acceptor are used. Alternatively, the method by Xia *et al.* [204] normalizes the FRET values for donor and acceptor concentrations as follows and produces even better results:


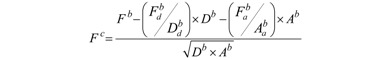
 (23)

Sensitized emission FRET measurements can be performed on wide field as well as on confocal microscopes with respective dichroics and filter sets. Major drawbacks of the sensitized emission method are: (i) the method is relatively laborious and requires extensive image processing; and (ii) the need for image processing increases all the noise in the images and might ultimately exceed the FRET signal in experiments where the FRET signal is weak to begin with. Thus, measuring and calculating the controls, applying the correction factors and calculating the final FRET signals may lead to substantial errors. The experiments become particularly tricky when this FRET approach is carried out on independently labeled fluorescent partners, *i.e.*, where the stoichiometry is not known or cannot be controlled and the concentrations of donor and acceptor differ significantly or even change during the experiments. Therefore, it is much easier to carry out such FRET experiments on intramolecular structural changes where the fluorescent labels are within the same molecule and the stoichiometry is well defined, *e.g.*, with Ca^2+^ sensors such as the Cameleons [166,205]. An excellent comparison of the methods used to quantify FRET in sensitized emission FRET was made by Berney and Danuser [206], and the interested reader is referred to that review. Nevertheless, despite these limitations, if no FLIM microscope is available, sensitized emission is a suitable and relatively inexpensive method to carry out FRET in dynamic live cell experiments, provided that the FRET signal is intense enough.

A major improvement of sensitized emission FRET, reducing the risk of cross-talk, noise and other unwanted effects to a minimum, is a further development of the aforementioned method and is named Spectral Imaging FRET. In this method, instead of detecting the donor and acceptor fluorescence intensities around λ_max_, the entire emission spectrum of both donor and acceptor is acquired upon donor excitation [207,208] in virtually the same way as in a fluorimeter. The advantage of this approach is obvious: by taking the distinctive shapes of the spectra into account, and any changes therein, information on FRET can be obtained with a much higher certainty and virtually devoid of any contamination from other channels. Spectral imaging therefore permits the researcher to directly evaluate the level of cross-talk because of direct excitation of the acceptor, and facilitates the accurate measurement and calculation of the true FRET signal [207].

#### 3.4.3. Fluorescence Lifetime Imaging Microscopy (FLIM)

Because the interpretation of intensity-based FRET measurements is challenging and limited by experimental conditions and possible artefacts, such as signal cross-contamination, the concentration and labelling efficiency of the fluorochromes, variations in excitation intensity and exposure duration, and photobleaching, researchers sought ways to overcome these limitation. Since the fluorescence lifetime τ is affected by energy transfer, but essentially insensitive to the aforementioned limitations, measuring the lifetime via FLIM provides essential information on FRET and ameliorates many limitations associated with intensity-based FRET. The first FLIM instrument was described as early as 1959 and was based on a frequency-domain microscope setup that only allowed single point measurements [209]. The first true FLIM imaging was reported by Wang *et al.* in 1989 [210]. FLIM was further developed independently by various groups, but Kusumi and co-workers were among the first to perform time-resolved fluorescence imaging in single cells [211].

FLIM is a technique that maps the spatial distribution of the lifetimes within microscopic images of fixed as well as living cells. As stated previously, fluorochromes are not only characterized by their excitation and emission spectra, but also by their unique lifetime. The fluorescence lifetime is the exponential decay in emission after the excitation of a fluorescent probe (see Equations 3 and 4). In the physical sense, the fluorescence lifetime τ is the time needed for the fluorescence intensity to decrease to 1/e (=1/2.71) of its initial value *I*_0_ and can generally be considered as the average time that the fluorochromes resides in the excited state. The fluorescence lifetime does not change upon intensity variations provided that sufficient signal is available to be detected. Furthermore, lifetime measurements are not dependent on the transmission efficiency of the microscope, the local concentration of the fluorochromes, the local excitation light intensity, or on the local fluorescence detection efficiency [11] and are generally insensitive to moderate levels of photobleaching [212]. However, even though the fluorescence lifetime is largely insensitive to the aforementioned factors, the fluorochrome’s lifetime is sensitive to its environment such as changes in pH, polarity, refractive index of the medium, temperature, to name but a few. Even though these phenomena need to be taken into account in some experiments, in others they provide the basis for mapping spatial variations of the lifetime in response to changes in the environment and thus may sensitively provide information on the biomolecule’s action or state *in vivo*. 

In FLIM-FRET, the presence of the acceptor causes energy transfer and as a result the donor looses its excited state energy faster than in the absence of the acceptor. Consequently, the donor fluorescence lifetime decreases concomitantly. Combination of FLIM and FRET allows the measurement of lifetime dynamics pixel-by-pixel and thus mapping of its spatial distribution to indirectly measure biomolecule concentrations, interactions between biomolecules, and conformational changes with a much higher accuracy than conventional FRET methods. The FRET efficiency (*E_FRET_*) may be calculated according to:


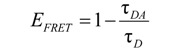
 (24)

where τ*_DA_* and τ*_D_* are the excited-state lifetimes of the donor in the presence and absence of the acceptor, respectively.

FLIM has been used in several applications, such as the analysis of protein-protein interactions with high temporal specificity, in ion concentration imaging as well as in measuring oxygen concentration and in various medical applications [213,214]. Thus, FLIM is an example of using fluorescence beyond emission intensity measurements and determining the spatial location or distribution of a molecule or protein, but rather FLIM allows examination of the protein micro-environment with high resolution. Although the FLIM instrumentation setup is more complex and expensive compared with other FRET detection methods, FLIM is the most accurate method for FRET measurements in live cell experiments, but equally cannot be considered a main-stream and trivial methodology. FLIM can be used in scanning confocal, multi-photon, or in wide field microscopes, and can be implemented in two ways: (i) either in the frequency domain, using sinusoidally modulated excitation light; or (ii) in the time domain, using pulsed excitation sources. Traditionally such pulsed lasers sources have been employed in multiphoton imaging and deliver discrete and consistent bursts of light to the sample. The lifetime of most fluorescence markers is in the order of a few nanoseconds, and most commercially available pulsed lasers deliver light in 12.5 nanosecond pulses, which provides a good window for lifetime imaging. Nonetheless, most fluorescence sources can be adapted for FLIM imaging provided that the light that reaches the sample is effectively modulated [215,216]. Generally, single-photon-counting is widely used in scanning FLIM, either time-correlated or time- and space-correlated, but a number of innovations have recently been introduced that allow faster data acquisition (discussed in [183]). In wide field FLIM, gated or modulated image intensifiers are generally used with CCD camera detection. This form of FLIM can be preformed in the time-domain mode, with pulsed excitation sources and gated or time-correlated single-photon counting or in the frequency domain with intensity modulating excitation and homodyne or heterodyne phase-sensitive detection [183]. Major advantages of wide field FLIM are that inexpensive light-emitting diodes (LEDs) can be used as excitation source, and the high acquisition speed, since no scanning is performed and consequently all pixels are acquired simultaneously. On the down side, because the emission is only sampled briefly, the maximum photon count is not reached, which translates in a limited accuracy in the lifetime measurement. Nonetheless, wide field FLIM might simplify the technique and reduce the costs to such an extent that FLIM can be routinely applied in various laboratories. 

**Figure 23:**
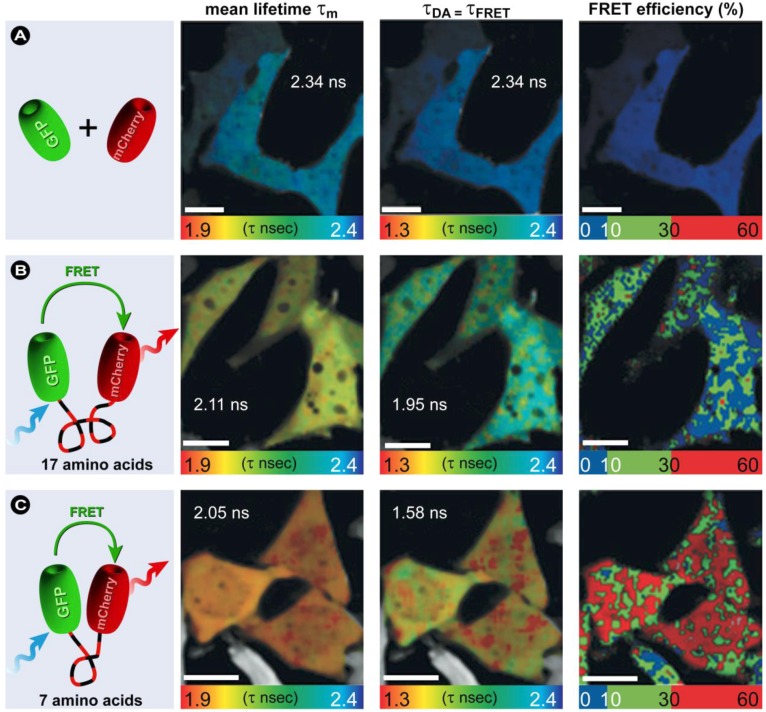
*In vivo* multiphoton FLIM-FRET measurements. Living HeLa cells co-expressing either unfused, free EGFP and unfused, free mCherry (**A**), or GFP-coupled directly to mCherry through a 17-amino-acid linker (**B**), or GFP-coupled directly to mCherry through a 7-amino-acid linker (**C**) were imaged by using a multiphoton scanning microscope. For each panel, the spatial distribution of the mean fluorescence lifetime (τ*_m_*) and of the fluorescence lifetime of the donor molecules interacting with the acceptor (τ*_DA_*) is shown throughout the cells. The FRET efficiencies were calculated for each pixel from Eq. 24 × 100%. Color scale shown covers the range of *E_FRET_* values from 0% to 60%. Bars, 10 μm. Adapted from [217] with permission. © 2007 John Wiley & Sons.

The combination of FLIM-FRET and TPE microscopy offers the same capability for life time measurements, but has the significant advantage of producing less scattering, increased spatial resolution and depth-sectioning. Other recent advances, including multi-parameter FLIM-FRET, acceptor fluorescence rise-time FLIM-FRET, and single molecule FLIM are discussed elsewhere [183].

FLIM-FRET has been used to address questions about protein conformational changes. For example, to study integrin-effector binding using α4 and β1 integrin in a quantitative manner using a GFP-mRFP FRET pair [218]. In this study FLIM-FRET was used to obtain information on how the conformational state of the receptor may determine the activity of anti-integrin molecules, by using assays capable of detecting integrin effector binding. Another example is the measurement of conformational changes in elongin C when co-expressed with elongin B, which were determined by the small increase in the intramolecular FRET efficiency using Cerulean and Citrine [219]. A ratiometric chloride indicator Clomeleon based on CFP and Topaz, a variant of YFP, was used for FLIM-FRET measurements as part of a study of neuronal development by monitoring intracellular chloride concentrations. It was found that the FRET signal correlated well with the neuronal development, with the Clomeleon lifetime indicating the concentration of chloride in the neurons [220]. Figure 23 shows an example of a FLIM-FRET experiment, with which Llères *et al*. demonstrate the effect of the linker peptide length between GFP and mCherry on the lifetime [217]. Notice that a seven amino acids (AA) linker displays a larger change in mean fluorescence lifetime (τ_m_) compared with the 17 AA linker. Furthermore, the FRET efficiency is significantly higher compared with the 17 AA linker (Figure 23B,C). In contrast, the negative control of free moving mCherry and GFP in Figure 23A show long lifetimes and zero FRET efficiencies, as expected. The differences in the FRET results are largely due to alterations in geometry and the increased distance between donor and acceptor.

Besides combining FLIM with FRET to study protein-protein interactions in living cells, FLIM is also effectively combined with various other techniques, including super-resolution microscopy using stimulated emission depletion (STED) [221], as further discussed below, in virus, yeast and bacteria detection [6], lifetime-based imaging of fingerprints in forensics [222], in microfluidic [223,224] and lab-on-chip devices [189], and other novel biosensors. Excellent reviews on the technical, theoretical, and biological aspects of FLIM can be found in [6,11,39,40,180,182,183,213,216,217,223,225,226,227]. Guides to easy quantitative FLIM analysis based on phasor analysis, in which the life time data is sine (S) and cosine (G) transformed into a spatial coordinate system, are given in references [228,229] and a modified form to allow time-gated fluorescence lifetime image analysis was recently developed in Hans Gerritsen’s group [230].

#### 3.4.4. Polarization Anisotropy Imaging

**Figure 24:**
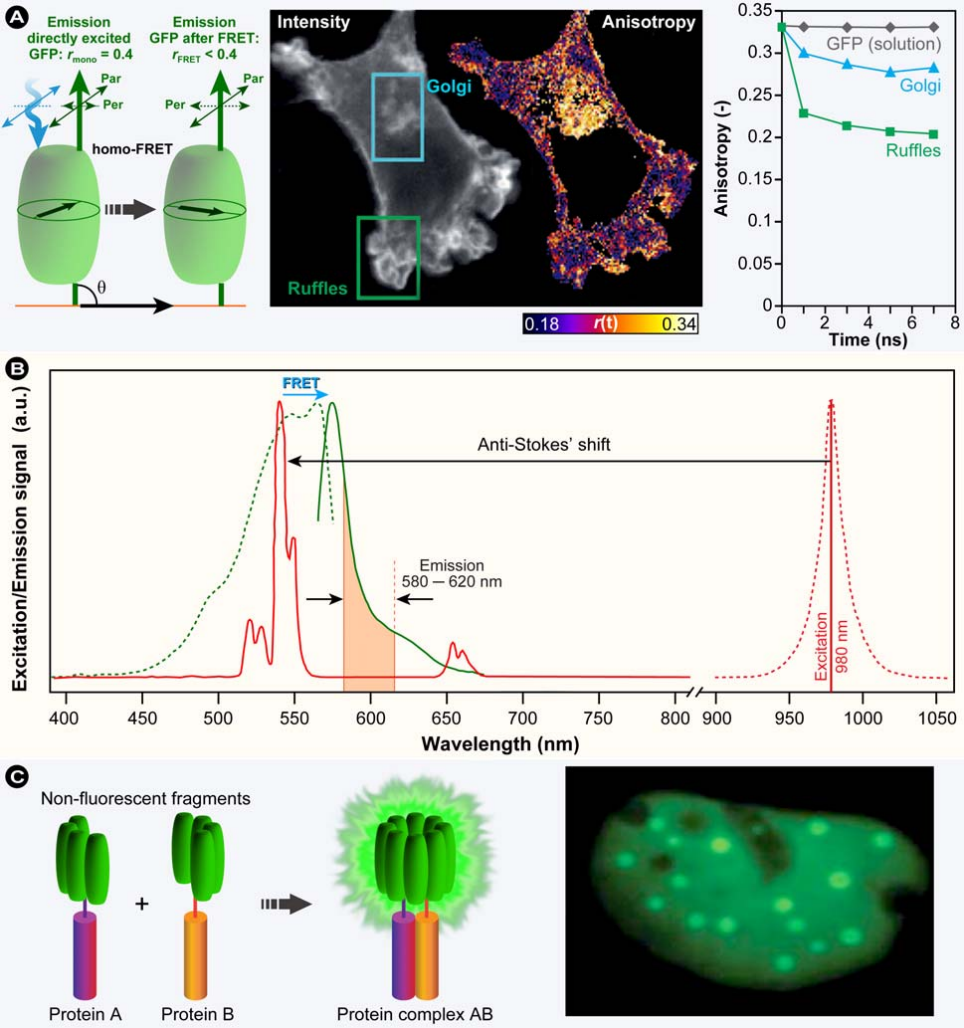
Advances in protein-interaction methods. (**A**) Schematic representation of homo-FRET in a FP dimer. Centre: Intensity and anisotropy micrographs of a NIH 3T3 cell expressing GPI-GFP. Right: Homo-FRET analysis (time-resolved) of two ROI indicated in the intensity image. (**B**) Upconversion-FRET: Excitation (dashed) and emission spectra (solid) for UCP (NaYF_4_:Er^3+^,Yb^3+^; red) and B-phycoerythrin (BPE; green).The emission spectrum of UCP (donor) overlaps with the excitation spectrum of BPE (acceptor) and energy-transfer excited emission of BPE can be measured at 600 nm and CW-laser diode excitation at 980 nm. (**C**) BiFC is based on the reassembly of two complementary non-fluorescent FP fragments, which is facilitated by interaction of proteins A and B, to yield a functional fluorochrome. The image shows the recruitment of R288P Maf and Sox proteins to subnuclear foci through dimerization and reconstitution of AB to yield fluorescence competent YFP. Partially adapted from references [66,232,233] with permission. © 2004 American Society for Microbiology. © 2008 New York Academy of Sciences.

Polarization anisotropy imaging is a method based on the measurement of fluorescence polarization [231] as introduced previously in §1.1.2. These measurements of fluorescence polarization offer particular advantages for high-contrast discrimination of FRET with FPs. The concept is based on the fact that excitation with polarized light is only possible in that part of the fluorochrome population that have absorption vectors aligned parallel to the polarization vector of the excitation light (Figure 24A), which is a process called “photoselection”. If the fluorochrome’s transition moment does not change, *i.e.*, the fluorochrome does not rotate, the major part of the fluorescence emission remains parallel to the excitation direction so that the fluorescence can be considered anisotropic in terms of polarization. 

The fluorescence anisotropy (*r*) is defined as the intensity-corrected difference between the emission parallel (*I*_par_) and perpendicular (*I*_per_) to the excitation polarization direction [8]:


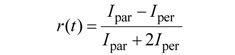
 (25)

The anisotropy will disappear if the molecules rotate during the nanosecond fluorescent lifetime. However, because of the relatively large size of FPs and their slow rotation, they do not display high fluorescence depolarization during the measurement. If FRET occurs between two FPs that are slightly misaligned, then the polarized fluorescence emission will emerge at a different angle (from the excitation vector), which is a projection of the FP’s rotation. The principal strength of this approach is that measuring fluorescence polarization parallel and perpendicular to the excitation is straightforward with high signal-to-noise ratios. Due to the fact that data can be acquired rapidly and minimal image processing is needed, this approach is well suited for applications in high-content screening. 

Polarization anisotropy measurements are not without disadvantages. Any direct excitation of the acceptor must be carefully avoided, because it decreases the donor signal and reduces the signal-to-noise ratio of the measurement. Anisotropy measurements require polarizers, which cause a significant reduction in the emission signal, and consequently higher concentrations of fluorochrome are needed compared with intensity-based measurements to obtain accurate results. In addition, although the polarization FRET technique is excellent in discriminating between the presence and absence of FRET, it is not a good approach for differentiating between strong and weak FRET. This approach is also susceptible to any polarization artifacts caused by the optical system used, *e.g.*, elements in the optical path such as beam-splitters, filters, mirrors. The polarization can be degraded in high numerical aperture objectives, so polarized FRET experiments should be limited to imaging with objectives with numerical apertures of 1.0 or less. As a consequence, polarization anisotropy imaging should only be applied after careful examination of the microscopic setup and control measurements to determine to what extent the optics influence the polarization. A successful application example of this technique is the ability to distinguish FRET between linked and unlinked Cerulean and Venus fluorescent proteins in living cells with a larger dynamic range than other approaches [169]. 

### 3.5. Homo-FRET versus Hetero-FRET

In previous examples of FRET, the donor and acceptor in a FRET pair are different species with distinct photophysical properties, in which energy transfer occurs irreversibly from the donor to the acceptor (hetero-FRET). As described in the above sections, in hetero-FRET, energy transfer can be measured either by monitoring quenching of the donor, the sensitized emission of the acceptor, or the changes in the fluorescence lifetime of the donor. Conversely, homo-FRET occurs between two identical molecules in which one is the donor that transfers the excited state energy to an identical molecule in close proximity, which acts as the acceptor. Such energy transfer between identical molecules neither induces any change in the donor fluorescence intensity nor fluorescence lifetime, since the population of excited state donor molecules is not actually reduced during energy transfer. Consequently, the only observable that truly changes as a result of the energy transfer is the fluorescence anisotropy, which is reduced in homo-FRET [8]. 

The main advantage of homo-FRET is the fact that only one fluorochrome is necessary for such studies and homo-FRET remains popular in membrane dynamics studies, such as studies on membrane protein clusters [234]. However, despite this obvious advantage, application of homo-FRET is significantly restricted because several factors frustrate the accurate rationalization of depolarization in homo-FRET, as recently pointed out by Loura and Prieto [235]: (i) back-transfer to the directly excited donor; (ii) transfer to any donor with possibly many transfer steps; and (iii) in addition to energy transfer, depolarization occurs through fluorochrome rotation and if these processes occur in the same timescale, these phenomena are not independent, but coupled. Such complicating factors are certainly obstacles for quantitative data analysis and the authors [235] also point out that many published reviews on the technicalities of homo-FRET fail to take these factors into consideration. For instance, Yeow and Clayton who extended commonly used formalisms for homo-FRET data analysis to allow for the occurrence of interoligomer energy transfer, which was critically needed because depolarization between overexpressed oligomeric proteins at high density in cell membranes may occur, do not take the aforementioned factors into consideration. Therefore, even though their model resolves one critical weakness, the mathematical description remains susceptible to error. Further modeling therefore seems necessary to consolidate homo-FRET data analysis and quantization.

### 3.6. Advances in Protein-Interaction Methods

#### 3.6.1. Upconversion FRET

In upconverting-FRET (UC-FRET), the donor commonly consists of a rare earth metal-containing upconverting chromophore (UCC) or nanoparticle (UCN) (see also §1.2.4) and a normal acceptor fluorochrome (“downconverting”). As in other FRET applications, an overlap requirement between the emission spectrum of the UCC and the excitation spectrum of the acceptor exists. A major advantage is that, because the UCC’s excitation spectrum is well separated from the excitation spectrum of the acceptor due to the large anti-Stoke shift, direct excitation of the acceptor does not occur (see Figure 24B). Further advantages include, sensitive acceptor emission detection, the excitation in the NIR/IR abolishes autofluorescence of endogenous fluorochromes, and the narrow donor emission band can be readily differentiated from the acceptor emission. All these features result in the detection of the sensitized emission against a dark background. Prolonged FRET sensing is particularly feasible with UCN, since their biocompatible coating acts as a shield, which results in low toxicity, high photostability and minimum photobleaching and photodamage. A major disadvantage is that such UCC cannot be genetically encoded or engineered and thus various methods that introduce the UCC or UCN into the cell will lead to some disruption of the cellular homeostasis.

UC-FRET as a technique is relatively new, but despite this a number of reports have appeared over the past few years that include applications in bio-assays, such as nucleic acid hybridization [236], ligand binding [237,238,239], enzyme activity [240], and competitive immunoassays [241]. Although UCN *per se* are now increasingly being developed for use in cellular and in small animal imaging (reviewed in [13]), FRET-based imaging assays are still scarce, not in the last place because *in vivo* labeling remains challenging with this technology and more work is required to develop UC-FRET for *in vivo* applications. Nonetheless, Jiang and Zhang reported an UC-FRET-based method based on attaching a siRNA-BOBO-3 complex to the surface of amino-group-modified silica/NaYF_4_:Yb,Er UCN to study intracellular release and biostability of siRNA in live cells [242].

#### 3.6.2. FRET Frustration

“Frustrated energy transfer” or “exciton blockade” was introduced and examined theoretically by Stefan Hell and co-workers [243]. The basic idea behind this multi-photon fluorescence process is that acceptors brought into the excited state through FRET cannot accept energy from a second excited state donor. Therefore, FRET can deliberately be abrogated, with recovery of the donor emission, by forcing direct excitation of the acceptor, thus “frustrating” FRET. In practice, such FRET abrogation would be most efficiently observed when periodically saturating the acceptor with a modulated light source and concomitant detection of the donor emission with phase-sensitive detectors, such as lock-in amplifiers [182]. Major advantages of this method include that, in contrast to non-resonant multiphoton absorption, this method does not require high local light intensities and a significant increase in resolution is predicted [243]. However, reports of applications are rare in the literature, most likely because recent research has shown that deviations from the theoretical deliberations occur in a number of fluorochromes–FRET has been shown to occur to acceptors that already reside in the excited state [244,245,246], which complicates matters. Energy transfer to ground and excited state acceptors is Förster allowed, because the acceptor does not change its spin and consequently singlet–singlet and singlet–triplet annihilation occurs. In common fluorochromes, such as cyanine- and rhodamine-dyes, which show considerable extinction in the visible spectrum, such annihilation processes cannot be completely avoided [247] and consequently, an energy-transfer blockade remains incomplete.

However, Tinnefeld *et al.* [248] recently showed that under certain circumstances, such a complete blockade or FRET frustration is possible when using radical anion states of fluorochromes as saturable dark states in “energy transfer blockade probes” (ETBPs) that consist of a donor surrounded by one to *n* acceptors (*n* = 0, 1, 2, ...). The authors furthermore demonstrate that these ETBPs exhibit a superlinear donor response because of acceptor saturation and this directly translates into resolution enhancement in confocal microscopy in all three dimensions. Despite these first positive results, FRET frustration as a technology remains immature and has partially been surpassed by a competing technology based on photochromic dyes and proteins.

#### 3.6.3. Photochromic FRET

One of the main limitations in FRET measurements involves the determination if FRET can be lifted, for instance by photobleaching the acceptor (see §3.4.1). Besides being irreversible, photobleaching-based FRET is generally neither suited for live cell experiments not for prolonged imaging. Other FRET techniques suffer from disadvantages such as variable donor to acceptor stochiometries (ratiometric FRET), expensive and elaborate instrumentation (FLIM, spectral, and polarization anisotropy imaging), phototoxicity, or the requirement for a large number of control images. The newly developed photo-switchable FPs conveniently allow a form of FRET called photochromic-FRET (pcFRET) in which a FRET-competent and incompetent state of the acceptor can be reversibly induced by illumination with a particular wavelength. pcFRET was first demonstrated using photo-switchable dyes [249,250] and subsequently further developed based on photo-switchable fluorescent proteins [250,251]. The major advantage of this method is the fact that it allows the continuous and prolonged measurement of FRET in living cells and the determination of FRET efficiencies with high accuracy. The sensitivity in pcFRET can significantly be enhanced by utilizing periodic switching with lock-in detection [250], which would be much more effective compared with the aforementioned FRET frustration.

Equal to FRET frustration, pcFRET aims to take the acceptor out of the equation , but in contrast to FRET frustration, pcFRET provides a more solid and reversible mechanism to switch the acceptor on and off, with fewer interference from alternate decay or transfer pathways. Therefore, it may be anticipated that the application of FRET frustration as a technique will remain limited, whilst pcFRET will become more widely used, especially because of the ease of use and the lack of elaborate equipment.

#### 3.6.4. Single-Molecule-FRET and Switchable-FRET

What traditional FRET techniques have in common are that they report the average behavior of a bulk population of molecules, albeit with temporal and spatial resolution. Events such as the dimerization of proteins to form a cell surface receptor or the measurement of the step size of a DNA helicase cannot be directly visualized or measured using these FRET techniques. In the 1990s, Taekjip Ha developed a technique called single molecule FRET (smFRET) in Simon Weiss’ lab. He used near-field scanning optical microscopy (NSOM) to simultaneously obtain dual color images and emission spectra from donor and acceptor fluorochromes linked by a short DNA molecule and thus provided proof of principle that conformational changes, such as rotations or distance changes, within single biomolecules may be detected with FRET involving a single pair [252]. Both donor and acceptor photobleaching were used to confirm the presence of FRET and to calculate the energy transfer efficiency. 

The main advantage of smFRET is the fact that events that are normally obscured in ensemble measurements, because they are canceled out due to random averaging, are now clearly visible and can be measured with high accuracy. A recent study by Biju and co-workers exemplifies this. This study showed that dimers of the epidermal growth factor receptor (EGFR) are continuously formed in cell membranes through reversible association of hetero-dimers [EGF(EGFR)_2_] and that the lateral propagation of EGFR activation takes place through transient association of a heterodimer with predimers [(EGFR)_2_] [253]. These results extent the previous findings by Gadella and Jovin using FLIM-FRET, which showed that in A431 cells a microclustering of EGFR occurs and that a subclass of receptors are present in a predimerized or oligomerized state [254]. Without smFRET imaging and by using bioconjugated quantum dots, this reversible receptor dimerization in the lateral activation of EGFR would have remained obscured. Nanoparticle-based smFRET (or better said single particle FRET) is increasingly being used for biological studies—predominantly with quantum dots, as exemplified by the study described above. It is even possible to produce smFRET between negatively charged nitrogen-vacancy centers in diamond nanoparticles as donors and organic dyes as acceptor [255], which has the additional advantage that nanodiamonds do not show any intermittent behavior.

smFRET has matured and become more reproducible over the past few years, predominantly because of advances in detector technology, novel fluorochromes, such as the aforementioned nanoparticles, and it has been the subject of intense research, especially to overcome some of the disadvantages associated with smFRET. A major advantage, however, is the fact that smFRET-capable systems can readily and cost-effectively be constructed with off-the-shelf components and either a confocal microscopy, or rather a total internal reflection (TIR) microscope, since smFRET temporal-spatial trajectories are predominantly acquired by imaging surface immobilized molecules. To obtain significantly more information, especially when multiple biomolecules are involved in a complex or multiple steps determine the sequential conformational change in a biomolecule during its normal biological activity, multiple labeling and consequently multiple smFRET would be required. Indeed, Ha and Hohng extended smFRET by using four fluorochromes to concomitantly observe the correlated motions of four arms of the Holliday junction, and to investigate the correlation of RecA-mediated strand exchange events at both ends of a synaptic complex via alternating laser excitation (ALEX) [256]. However, the complexity of the data processing scales with the number of concomitantly used fluorochromes, and therefore there is a limit to what is feasible and practical in multiple smFRET. Furthermore, smFRET *in vivo* is inherently difficult because of autofluorescence and because FP-labels do not have advantageous photophysical properties for smFRET. Kapanidis *et al.* aimed to resolve some of these issues and turned to photo-switchable FPs, which allows FRET between a single donor and multiple, spectrally identical photo-switchable acceptors [257]. This “switchable-FRET” method reduces both the experimental and analytical complexity and most likely introduces scalability with regard to monitoring multiple distances simultaneously. 

A good practical guide to smFRET can be found in reference [258] and a general overview of single molecule techniques in reference [259]. A general and systematic catalog of FRET techniques, dyes and fluorescent proteins, adapted to various imaging systems, including some new approaches for implementation can be found in more specialized reviews [39,40,121,124,138,182,225,226,227,260,261,262,263,264].

#### 3.6.5. Bimolecular Fluorescence Complementation

Finally, an alternate approach to measuring protein-protein interactions requires a brief introduction. Bimolecular Fluorescence Complementation (BiFC) is based on the reassembly of a functional fluorescent protein label from non-fluorescent fragments of FPs fused to proteins of interest whose interaction facilitate the label’s reassembly (Figure 24C). Biochemical complementation of fragments has been known for subtilisin-cleaved bovine pancreatic ribonuclease since 1958 [265], but truly conditional fluorescence complementation was first shown by Kerppola et al. with YFP fragments fused to either the basic-region leucine zipper (bZIP)-domain or Rel-family proteins [266]. 

BiFC offers some advantages over classical FRET-based assays, since these assays only report on interactions by a small number of interacting proteins and therefore require overexpression of interacting partners to obtain sufficient sensitivity. Such overexpression is no reflection of the real cellular state and might even disrupt normal cellular processes or cause formation of non-native complexes; all factors that might perturb results. Furthermore, since in BiFC a functional fluorochrome is formed from non-fluorescent fragments, BiFC is less prone to interference from changes in the fluorescence intensity or lifetime due to interaction unrelated environmental factors than FRET-based assays. In addition, BiFC allows the simultaneous study of multiple interaction partners for a given protein of interest with multiple combinations of fusion proteins.

Conversely, the major disadvantages of BiFC are that: (i) BiFC cannot visualize protein interactions in real time, because of the high stability of the protein complex and the long time necessary for fluorochrome maturation (~8 h for the reconstitution of YFP [266]); and (ii) because of the high complex stability, transient or dynamic interactions in which partners associate for a particular time and subsequently dissociate cannot be imaged. In this sense, FRET is more versatile and allows the imaging of dynamic processes. Detailed technical reviews on BiFC can be found in references [267,268,269,270], whilst excellent overviews were recently provided by Kerppola [271,272].

### 3.7. Combination of FRAP and FRET

**Figure 25:**
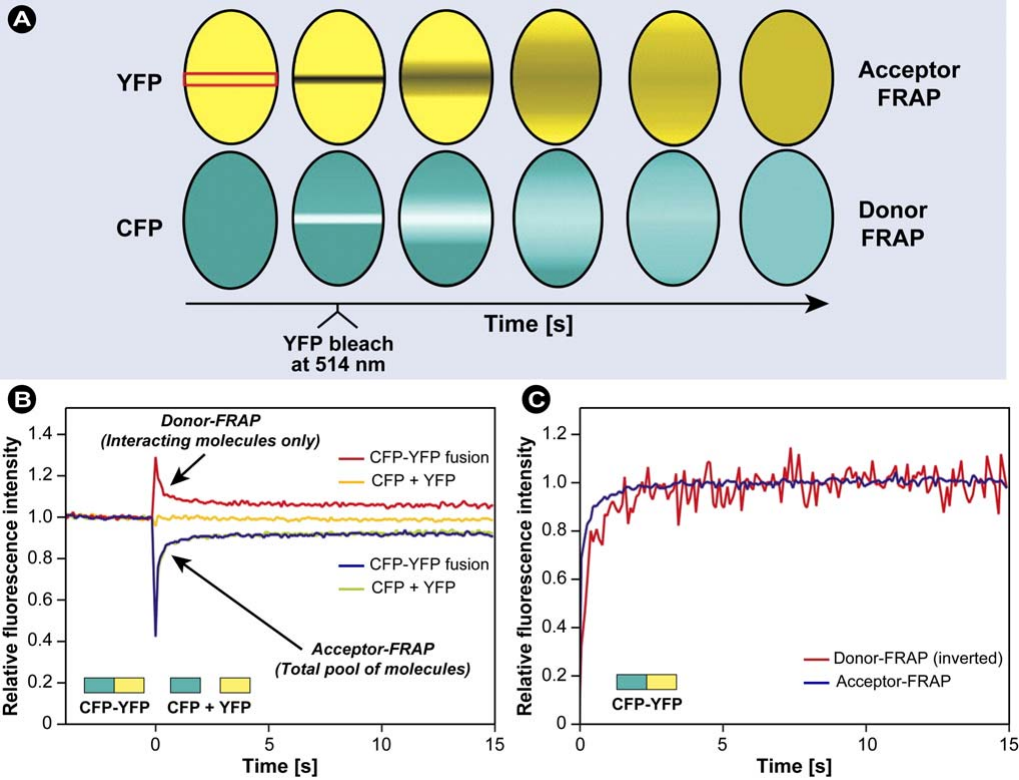
Simultaneous FRAP and FRET measurements to separately determine the mobility of interacting and noninteracting CFP- and YFP-tagged proteins in a single cell nucleus. (**A**) Schematic representation of the method. A 100 ms high-intensity bleach pulse at 514 nm is applied to irreversibly photobleach YFPs in a narrow strip spanning the nucleus. Redistribution of YFP and CFP fluorescence is recorded at 100 ms intervals at 458 nm. Donor (CFP) emission (increased because of unquenching as a result of acceptor [YFP] bleaching) represents the mobility of interacting molecules only (donor-FRAP). Acceptor emission represents the total pool of YFP-tagged molecules irrespective of interaction (acceptor-FRAP). (**B**) Graph showing CFP and YFP fluorescence intensities in the bleached strip plotted against time. Experiments were performed in Hep3B cells expressing CFP-YFP fusions (red line indicates CFP fluorescence [donor-FRAP], and blue line indicates YFP fluorescence [acceptor-FRAP]), or in Hep3B cells expressing separate CFPs and YFPs (yellow line indicates CFP, and green line indicates YFP; *n* = 30). (**C**) Inverted donor-FRAP (red line) and acceptor-FRAP (blue line) plotted against time, showing similar kinetics. The curves were normalized by calculating I_norm_ = (I_raw_ − I_0_)/(I_final_ − I_0_), where I_0_ and I_final_ are the fluorescence intensities immediately after the bleach and after complete recovery, respectively.

Although energy transfer techniques sophisticatedly allow the measurement of protein interactions in living cells, they provide no information on the mobility of interacting molecules. Innovatively, this was achieved in Adriaan Houtsmuller’s group by combining FRAP and FRET to yield a technique that made exactly such investigations possible [273,274]. Unlike the aforementioned FRAP methods in which a particular spot is photobleached, the author use what they call strip-FRAP in which a narrow strip spanning the nucleus is bleached (Figure 25A). To obtain information on the mobility of associated proteins, FRET-donor (CFP) and FRET-acceptor (YFP) fluorescence are simultaneously acquired over time after irreversibly photobleaching the acceptor in a defined sub-region of the nucleus [273]. As shown in Figure 25, the donor fluorescence increases after acceptor photobleaching only to subsequently decrease as a result of diffusion (donor-FRAP), which reflects the mobility of the interacting molecules only. On the contrary, acceptor fluorescence redistribution after acceptor photobleaching (acceptor-FRAP) provides information on the mobility of the total molecule pool, both interacting and non-interacting. Furthermore, Houtsmuller *et al.* point out that comparison of donor-FRAP and acceptor-FRAP curves makes it possible to distinguish between the mobility and immobilization of subpopulations of interacting and non-interacting proteins.

The authors established and validated the method in Hep3B cells expressing either a CFP-YFP fusion protein or separate CFPs and YFPs (Figure 25B,C). The method of FRAP-FRET essentially works as follows:

1) A narrow strip across the nucleus was scanned at 458 nm excitation at 100 ms intervals and low laser power, and donor (CFP) and acceptor (YFP) signals were both acquired.
2) After 40 scans, specifically YFP was photobleached with a high-intensity 100 ms pulse at 514 nm.
3) Acquisition of the acceptor and donor signals in the bleached strip was resumed at 458 nm, but at considerably lower laser intensity.

Artifacts from YFP or CFP’s intrinsic fluorescent properties could be excluded, since co-transfected cells with separate YFPs and CFPs constructs did not show a donor-FRAP signal (Figure 25B). Plotting the inverted donor-FRAP and acceptor-FRAP in one graph revealed that similar kinetics were involved (Figure 25C).

### 3.8. Super-Resolution and FRET Microscopy

FRET imaging is a tool to measure interactions between fluorescently labeled molecules. It is an indirect method to visualize such interactions beyond the limits of classical microscopes (diffraction limit) and as stated earlier, FRET functions well at distances below 10 nm. Conventional microscopes are limited in their resolution because of diffraction of the imaging light and the aperture of the optics used. 

As discussed in § 1.2.2, Ernst Abbe described in 1873 that the smallest resolvable distance between two points using a conventional light microscope cannot be smaller than half the wavelength of the imaging light (λ/2NA; NA = numerical aperture) [48]. Abbe’s limit essentially prevents imaging below ~200 nm laterally. Thus the spatial resolution is limited by the wavelength as a result of the refractive indices of the media through which the beam passes and the cone angle of the focused light (Figure 7A), which can be significantly improved by using shorter wavelengths. However, in biological systems, the use of shorter wavelengths is restricted by a greater propensity to damaging effects, ROS formation and increased light scattering. 

**Figure 26:**
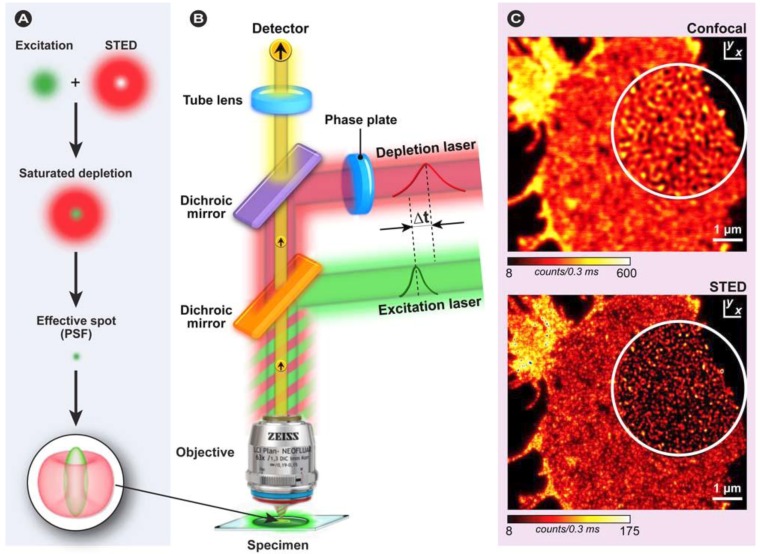
Principle of stimulated emission depletion (STED) microscopy. (**A**) STED is based on shrinking the excitation focal spot by depleting the outer excited state fluorochromes through stimulated emission with a doughnut-shaped STED beam of red-shifted and Δt time-shifted light (**B**). In essence the excitation PSF is combined with the PSF of the STED depletion laser (**B**) to produce a resultant PSF that is smaller than the diffraction limit of light. (**C**) Ultra-high resolution nanopattern distribution of the antibody-tagged SNARE protein SNAP-25 on the plasma membrane of a mammalian cell imaged with confocal and STED microscopy. The encircled areas show linearly deconvolved data. STED microscopy provides a substantial leap forward in the imaging of protein self-assembly; here it reveals for the first time that SNAP-25 is ordered in clusters of <60 nm average size. Part C adapted from [288]. © 2006 IOP Publishing Ltd.

For more than a century, Abbe’s diffraction limit was the *status quo*, until the optical diffraction barrier was broken utilizing the evolving knowledge on photoluminescence and consequently choosing circumstances in which this limit no longer was valid. Since then, there has been a dramatic technological development of various super-resolution techniques that are based on different approaches. What these methods have in common is that they offer spatial resolutions beyond the diffraction limit of conventional microscopes by exploiting nonlinear phenomena, utilizing switchable fluorochromes and localizing individual fluorochrome molecules with high spatial accuracy. Such techniques include stimulated emission depletion (STED, [275]), GSD (ground-state depletion) [276], structured illumination approaches (SIM, [277]), photo-activation localization microscopy (PALM, [278]), stochastical optical reconstruction microscopy (STORM, [279,280]) and others. As indicated previously, these techniques can be broadly divided into two categories [281]: (i) techniques that utilize narrowing of a fluorescent spot by modulation of transitions between two molecular states, which includes STED (notice the extreme increase in resolution in Figure 26), GSD, and saturated structured illumination microscopy (SSIM [282]); and (ii) techniques that pursue single molecule detection and establishment of their precise location by repeated switching of a limited number of fluorochromes in the total pool from which a super-resolution image can be reconstructed. The latter group of techniques includes STORM and PALM and these techniques would not be possible without the use of fluorescent proteins with irreversible or reversible light-induced photo-transitions. Recently, STED microscopy was extended and made suitable for two-photon excitation by combining a short-pulse laser source for two-photon excitation and a continuous-wave (CW) laser source for resolution enhancement with an achievable 4–5.4-fold improvement over the diffraction barrier, as described by Moneron and Hell [283]. This combination was subsequently modified by Bianchini and Diaspro, who showed that it is possible to achieve CW-TPE with the very same beam used for STED-CW on a commercially available system [284].

Excellent overviews on both photo-activatable/convertible proteins and their utilization in super-resolution microscopy were recently published by Stepanenko *et al.* [281] and Patterson *et al.* [285]. What should not be forgotten, however, is that in essence imaging beyond the diffraction limit with the current techniques is strictly limited to photoluminescence-based microscopy and consequently, in normal white light microscopy, Abbe’s limit still stands firm. Nonetheless, recent developments in nanotechnology aim to achieve white light imaging beyond the diffraction limit using nanolenses and a significant step towards such a system was recently achieved by Wang and co-workers [286]. 

The question arises whether these techniques will supersede FRET and make it obsolete by directly offering the spatial resolution to visualize protein interaction partners. In a recent review, Grecco and Verveer address this issue in-depth and the interested reader is referred to their excellent analysis [287]. Currently the resolution of these super-resolution techniques is not quite at the level of FRET yet. 

In addition, FRET has proven its practicability in live cell experiments and allows imaging and measurement of interactions in dynamic cell scenarios. What needs to be taken into account is the fact that with the majority of the currently available super-resolution technologies, fast dynamic processes cannot be imaged, because the acquisition time is too high and takes up to several minutes. The fastest acquisition is presently achieved with the Vertico Spatially Modulated Illumination Microscope (Vertico-SMI) developed by Chistoph Cremer. Recently, it was shown that a complete 3D SMI data stack could be acquired in a few seconds when imaging a tet-operator repeat insert in living U2OS cells [289]. In most cases, super-resolution approaches require bright fluorescent dyes and high excitation intensities, further limiting their use in live cell imaging experiments. Finally, FRET has been used to visualize small conformational changes within molecules and FRET-based indicators have been proven to be very useful sensors to measure ion concentrations with high spatial and temporal resolution [166,205]. However, it seems feasible that super-resolution techniques can be combined with FRET measurements. Sensitized emission and acceptor bleaching approaches are methods that could benefit from smaller imaging volumes such as in the case of STED or from structured illumination approaches. Thus, increasing the image acquisition speed for live cell FRET imaging will be a major challenge.

**Figure 27:**
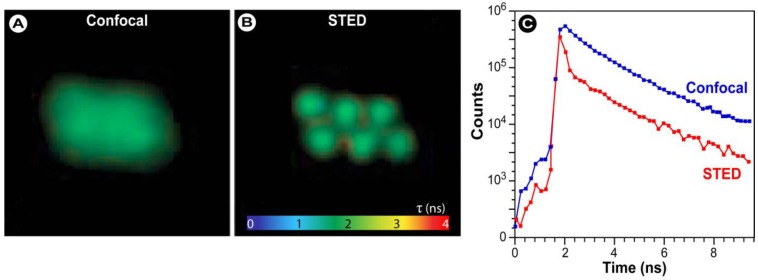
STED-FLIM in fluorescent 200 nm nanobeads (Molecular Probes). Intensity merged fluorescence lifetime images (x–y plane), recorded in **(A**) confocal and **(B) **STED mode with the doughnut-shaped STED beam. **(C)** Spatially integrated fluorescence decay curves obtained from confocal and STED images. Reproduced from [221] with permission. © 2008 Optical Society of America.

That a further development of super-resolution microscopy for nanoscale interaction imaging is not the realm of imagination is demonstrated by recent research by Auksorius *et al*. [221] who provided proof of principle with fluorescent nanobeads (Figure 27) that STED-FLIM is feasible, and the use of STED in combination with fluorescence correlation spectroscopy (FCS) by Eggeling’s group [290] to observe transient formation of cholesterol- and cytoskeleton-modulated lipid complexes in living cells. 

Excitingly, Deng *et al.* [291] show that the use of STED in combination with FRET couples in nanobeads is not only feasible, but that a threefold increase in resolution without the need to increase the intensity of the depletion beam can be achieved with this FRET-assisted stimulated emission depletion (FASTED) microscopy over conventional STED. Such advancements using controllable circumstances in model systems provide the basis for the further development of these techniques for *in vivo* applications.

## 4. Concluding Remarks

The significance of imaging-based methodologies is underscored by the fact that the major part of discoveries in life sciences over at least the past 50 years were based on imaging biological and pathological processes. Not in the least, the advanced fluorescence microscopic techniques described in this review all had a major impact on cell biological research and (bio)medicine. Complementary evolutionary progress in fluorescence microscopy and fluorochrome development, from newly synthesized dyes to engineered FPs and nanoparticles, with a constant technological exchange and traffic between these, have led to such a diverse number of fluorescence-based research tools. In particular the significant expansion of the FP-color palette beyond the classical green fluorescent protein and improvements made through genetic engineering to increase brightness or produce narrower spectral band-widths—predominantly developed in Roger Tsien’s lab [168,292,293,294], whilst others were discovered in other aquatic animals by researcher such as Mikhail Matz and Sergey Lukyanov [295]—have propelled many of the aforementioned techniques into the mainstream. Nowadays, functional insight in cellular processes that was previously the mainstay of analytical biochemistry and could only be observed on gels and blots can now directly be visualized and also be analyzed in the living cell. However, even though techniques such as FRAP, FLIP, and FRET have become more common place in life science research, they are certainly not trivial methodologies with many possible pitfalls, some of which are obvious, whilst others are more obscure. Therefore, as described in this review, fluorescence microscopic techniques require profound knowledge of fluorescence and its idiosyncrasies, necessitate careful planning of the experiment and the essential controls, and the results need to be seen in perspective of possible methodological pitfalls to avoid drawing erroneous biological or medical conclusions. Nonetheless, the conscientious and able researcher will master these techniques and develop them further, since there is still room for improvement, diversification, and innovation. 

Continuing developments in the fluorochrome-field will unquestionably include new probes with enhanced characteristics: FPs with narrower bandwidths enabling imaging with multiple FRET-pairs, novel fluorescent nanoparticles with reduced mean sizes devoid of fluorescence intermittency, probes with enhanced stability and brightness, or multi-modal probes that unite multiple imaging options in the same nanoparticle [1]. Fluorescent proteins with improved properties, such as enhanced maturation, increased photo-stability and fluorescence intensity will certainly advance future applications of various FRET-based methods. Furthermore, microscope technique evolution will lead to high-content and automated imaging that enables at least medium-through-put screening of bio-active components, improved algorithms to spectrally unmix signals or process spectral data with high accuracy, nanolenses that will enable combination of fluorescence imaging with white light nanoscopy, and finally increased acquisition speed in a myriad of microscopic and super-resolution techniques will enable future researchers to image dynamic processes in real-time with unsurpassed resolution.

Currently, the main challenge in FRET imaging remains to establish methods that allow the imaging of multiple fluorescent protein-based FRET couples in the same cell to obtain multiple parameter information concomitantly. This would constitute a significant leap forward in cell biological research. However, the main bottle-neck lies in the fact that the currently available couples have substantial overlap of excitation and emission spectra precluding parallel detection. One approach would be to move away from fluorescent proteins and use luminescent nanoparticles. Such a strategy would mean giving up the genetic advantage of direct expression in living cells, but would offer the benefit of using fluorescent labels with high photo-stability, narrow spectra, which would allow excellent separation of signals, and photo-activation and switchability. One major additional advantage that nanoparticles, and even organic dyes, hold over fluorescent proteins is their fluorescence brightness (~120 and ~490 collected photons/molecule for Dronpa and EosFP [161] *versus* ~6,000 collected photons/molecule for Cy5 and Cy5.5 [160]). However, in quantum dots, the extremely broad absorption spectrum basically precludes many FRET applications, even though the emission spectra are narrow and well separated. Therefore, successful combinations with organic dyes or the use of particles, such as nanodiamonds, seem two ways forward if engineering new QDs with improved spectral properties cannot be achieved. Nanoparticles for FRET and super-resolution microscopy were comprehensively reviewed by Tian *et al.* [296]. Nonetheless, engineering of FRET pairs with improved spectral separation and higher quantum yields would be highly desirable to obtain multi-parameter FRET. First steps towards this goal have recently been achieved with blue FP Sirius allowing dual-FRET imaging of Ca^2+^ and caspase-3 activation in apoptotic cells [297] and yellow LSS FP mAmetrine for accurate measurement of the delay between the onset of caspase-3 activity in the cytoplasm and in the nucleus during apoptosis [262].

As described previously, in switchable-FRET [257], such multiplexing capabilities are already used for multiple single molecule tracking and extension of this technique to include multiple donors with multiple switchable acceptors would be a significant step toward detecting multiple FRET-pair signals concomitantly in living cells. Another major challenge is to move away from pure *in vitro* biochemistry in which processes within biomolecules, *e.g.*, helicase action or biological motors, are measured, but rather to perform smFRET in living cells. This road is littered with obstacles, since tracking of labeled individual interacting biomolecules in a biomolecule soup is inherently difficult, *in vivo *labeling even with FPs has its quirks, and suitable fluorochromes for this purpose have to be developed. Wenigner and Sakon, recently developed a FRET technique which might circumvent these difficulties by microinjecting pre- and site-specific labeled recombinant SNARE proteins with a FRET donor and acceptor into living cells [298]. They observed, utilizing this method, that individual SNARE proteins, which are a class of proteins involved in cell membrane fusion, rapidly incorporate into folded complexes at the cell membrane. Microinjection as a technique is certainly not new and always carries the risk that because of the relatively large pipette tip size (~µm) and high injection volume, incomplete sealing of the plasma membrane, loss of turgor, and damage to the cell is induced [299]. Femtosyringes [299] and more the recently developed Attosyringe [300] or the carbon nanotube-based cell nanoinjector [301] would certainly improve the method developed by Weninger, since this would lead to significantly fewer perturbations.

Furthermore, it needs to be pointed out that FRET is often referred to as a “molecular ruler”, which suggests that the method can provide information on the precise distance between two labeled molecules. This is not so, since FRET is only a proximity assay that may be prone to low and fluctuating signal intensities, photobleaching, and a limited distance of ~10 nm beyond which no FRET occurs. These factors prevent FRET from providing exact information about the distance between two labeled molecules. True molecular ruler techniques exist, as developed in Paul Alivisatos’ group utilizing plasmon coupling to monitor distances between single pairs of gold and silver nanoparticles [302]. With this technique, prolonged and continued distance measurements up to 70 nm can be achieved, devoid of blinking and photobleaching, and recently protocols for calibrating experimental procedures to improve their performance were also published [303]. Alternatively, a method based on X-ray scattering interference between two gold nanoparticle probes for distance and distance distribution measurements was recently introduced by Harbury and co-workers [304].

To end with, super-resolution microscopy is only possible because the developed techniques use some property of photoluminescence to shrink the imaging spot or signal beyond Abbe’s diffraction limit. However, even in white light optical microcopy, Abbe’s limit is tumbling, predominantly because of the enormous advances made in nanotechnology over the past decade. Super-resolution optical imaging using nanolenses might see its breakthrough in the next few years and when this happens not only will Abbe’s diffraction limit become fully obsolete, but it will be interesting to see what modification will be made to the advanced fluorescence microscopic imaging techniques described in this review. The future is bright!
